# The first mitogenomic phylogenetic framework of *Dorcus* sensu lato (Coleoptera: Lucanidae), with an emphasis on generic taxonomy in Eastern Asia

**DOI:** 10.1186/s12862-024-02225-2

**Published:** 2024-05-21

**Authors:** Muhammad Jafir, Liyang Zhou, Yongjing Chen, Xia Wan

**Affiliations:** Department of Ecology, School of Resources and Environmental Engineering, Anhui University, 230601 Hefei, Anhui China

**Keywords:** Mitogenome, Lucanidae, *Dorcus*, Phylogenetics, Stag beetles, Taxonomy

## Abstract

**Background:**

*Dorcus* stag beetles in broad sense are one of the most diverse group in Lucanidae and important saproxylic insects playing a crucial role in nutrient recycling and forest biomonitoring. However, the dazzling morphological differentiations have caused numerous systematic confusion within the big genus, especially the puzzlingly generic taxonomy. So far, there is lack of molecular phylogenetic study to address the chaotic situation. In this study, we undertook mitochondrial genome sequencing of 42 representative species including 18 newly-sequenced ones from Eastern Asia and reconstructed the phylogenetic framework of stag beetles in *Dorcus* sensu lato for the first time.

**Results:**

The mitogenome datasets of *Dorcus* species have indicated the variable mitogenomic lengths ranged from 15,785 to 19,813 bp. Each mitogenome contained 13 PCGs, 2 rRNAs, 22 tRNAs, and a control region, and all PCGs were under strong purifying selection (Ka/Ks < 1). Notably, we have identified the presence of a substantial intergenic spacer (IGS) between the *trnA*^*ser (UCN)*^ and *NAD1* genes, with varying lengths ranging from 129 bp (in *D. hansi*) to 158 bp (in *D. tityus*). The mitogenomic phylogenetic analysis of 42 species showed that Eastern Asia *Dorcus* was monophyletic, and divided into eight clades with significant genetic distance. Four of them, Clade VIII, VII, VI and I are clustered by the representative species of *Serrognathus* Motschulsky, *Kirchnerius* Schenk, *Falcicornis* Séguy and *Dorcus* s.s. respectively, which supported their fully generic positions as the previous morphological study presented. The topology also showed the remaining clades were distinctly separated from the species of *Dorcus* sensu lato, which implied that each of them might demonstrate independent generic status. The Linnaeus nomenclatures were suggested as *Eurydorcus* Didier stat. res., *Eurytrachellelus* Didier stat. res., *Hemisodorcus* Thomson stat. res. and *Velutinodorcus* Maes stat. res. For Clade V, IV, III and II respectively.

**Conclusion:**

This study recognized the monophyly of *Dorcus* stag beetles and provided a framework for the molecular phylogeny of this group for the first time. The newly generated mitogenomic data serves as a valuable resource for future investigations on lucanid beetles. The generic relationship would facilitate the systematics of *Dorcus* stag beetles and thus be useful for exploring their evolutionary, ecological, and conservation aspects.

**Supplementary Information:**

The online version contains supplementary material available at 10.1186/s12862-024-02225-2.

## Background

*Dorcus* stag beetles are a diverse group in Lucanidae with over 150 species that have been described worldwide, and about 80 taxa of them are found in East Asia [1–6]. Like most stag beetles, the *Dorcus* members are well-known for their robust body shape, and exaggerated mandibles of males that resemble sword or knife shapes [3, 7, 8]. Some of them have been recognized as male trimorphism and thus as a good model for studying the evolution of sexually selected traits and behavior [9, 10]. Ecologically, stag beetles can serve as important bioindicators of forest health and ecosystem quality, as they are saproxylic in nature and their larvae feed on dead and decaying wood thus adding the organic matter back to the soil. Moreover, the diversity and population of stag beetles can provide information about the environmental conditions of an ecosystem [11–13]. Kuranouchi et al. [14] reported that during feeding, larvae of the *Dorcus rectus* reduced the acetylene into ethylene, thus playing a vital role in nitrogen fixation. A few species, such as *Dorcus binodulosus* in Japan and *Dorcus antaeus* in China, have been on the local conservational list due to their sensitivity to environmental changes [15].

Despite the peculiarity of these beetles, the taxonomy and phylogeny of *Dorcus* have long been unclear or chaotic situation. MacLeay (1819) established the genus *Dorcus* based on the male morphological traits and hence named *Dorcus* sensu stricto (*Dorcus s.s.* MacLeay) [16, 17]. Successively, some scholars added the species in *Dorcus* s.s. MacLeay. Later, Arrow (1950) packed 27 genera in Lucanidae into *Dorcus* MacLeay and formed the “*Dorcus* Arrow; also called *Dorcus* sensu lato (*Dorcus* s.l.)” with the opinion that male morphological characters are more dynamic (greatly varied) while female morphology is relatively stable and suitable for classification [18]. After that, different classification was presented in different catalogues or monographs [7, 19–23]. Fujita [2] largely accepted the reinstatements in the part work of Arrow, *Dorcus* s.l. contains *Dorcus* s.s. MacLeay, *Serrognathus* Motschulsky, *Macrodorcas* and *Hemisodorcus*. Later, Huang and Chen [3] based on male morphology and genital characteristics, indicated that *Serrognathus* Motschulsky, *Falcicornis* Séguy, and *Kirchnerius* Schenk were independent genera. So far, the systematics of this genus remained debatable and might be influenced by coevolution and phenotypic plasticity [24, 25]. Although, many attempts have been made to discuss the phylogenetic relationships of the genus *Dorcus* by using monogenic and polygenic genes as molecular data sets. Hosoya et al. (2001) investigated the genetic variation of 16 S rRNA gene in *Ceruchus lignarius* and *Dorcus rectus rectus*, intraspecific, intraspecific and interspecific relationships were discussed [26]. Hosoya and Araya (2005) supported the monophyly of *Dorcus velutinus* group using mitochondrial 16 S rDNA sequences as evidence [27]. However, their research only explored the complex species of *Dorcus*, and not involve the classification of *Dorcus* at the genus level. Hosoya (2003) carried out a phylogenetic analysis of *Dorucs* (MacLeay, 1819) and its two related genera (*Prosopocoilus* (Hope, 1845) and *Prismognathus* (Motschulsky, 1860)) based on *COI* gene [28]. Although their results supported the monophyly of *Dorcus*, however, its phylogenetic analysis found that the species in the genus *Dorcus* formed multiple lineages, but it still defined it as *Dorcus*, and their findings were questioned. Moreover, the genetic attempt has not been carried out to resolve the controversies in *Dorcus* taxonomy at generic level.

Recently, mitogenomics has revolutionized the field of taxonomy that uses the mitochondrial genome data sets. Because the mitochondrial genome exhibits a range of advantageous features including rapid evolutionary dynamics, maternal inheritance, limited recombination incidence, low molecular weight, and conserved gene order [34]. Such attributes facilitate broad comparisons for many animals, making it a valuable tool for phylogenetic reconstruction and as a model for genome evolution [34–37]. Moreover, the mitogenomes of insects generally contain 13 protein-coding genes (PCGs), two ribosomal RNAs (rRNAs), 22 transfer RNAs (tRNAs), and a non-codding region also known as the control region [29, 38]. The arrangement of genes within the insect mitochondrial genome is highly conserved across different species. This conserved gene order allows for the identification and comparison of homologous genes, aiding in the alignment and analysis of mitogenomic sequences. Various biologists analyzed the mitogenome data set through bayesian inferences (BI) and maximum likelihood (ML) methods and evaluated as a powerful tool to reconstruct the phylogenetic relationship among insects [39, 40]. IB analysis provides a statically robust framework to estimate evolutionary relationships. Its flexibility and ability to integrate phylogenetic uncertainty into downstream analysis make it dispensable for studying evolutionary relationships and processes in diverse taxa [41]. Both BI and ML analysis are essential for phylogenetic studies as they are complement each other. BI allows for the incorporation of prior knowledge and provides an estimation of uncertainty [41], while ML analysis offers a computationally efficient method to infer phylogenetic trees and evaluate alternative evolutionary models, collectively enhancing the accuracy and reliability of phylogenetic reconstructions [42]. Recently, similar methods have been employed for the establishment of phylogenetic relationships among various lucanid taxa, and completely resolved topology characterized by substantial nodal support have been assessed [31, 33, 43].

Therefore, the mitogenomic-based phylogenetic investigation has been conducted to reconstruct the phylogenetic relationship among different species of the genus *Dorcus* using BI and ML analysis. This attempt has successfully resolved the controversies in *Dorcus* because this genus has long been considered relatively debatable. Additionally, genomic organization, composition, and evolutionary rates in the mitogenome of 18 newly identified *Dorcus* species have been documented.

## Results

### Mitogenome Composition and Organization

**Fig. 1:**
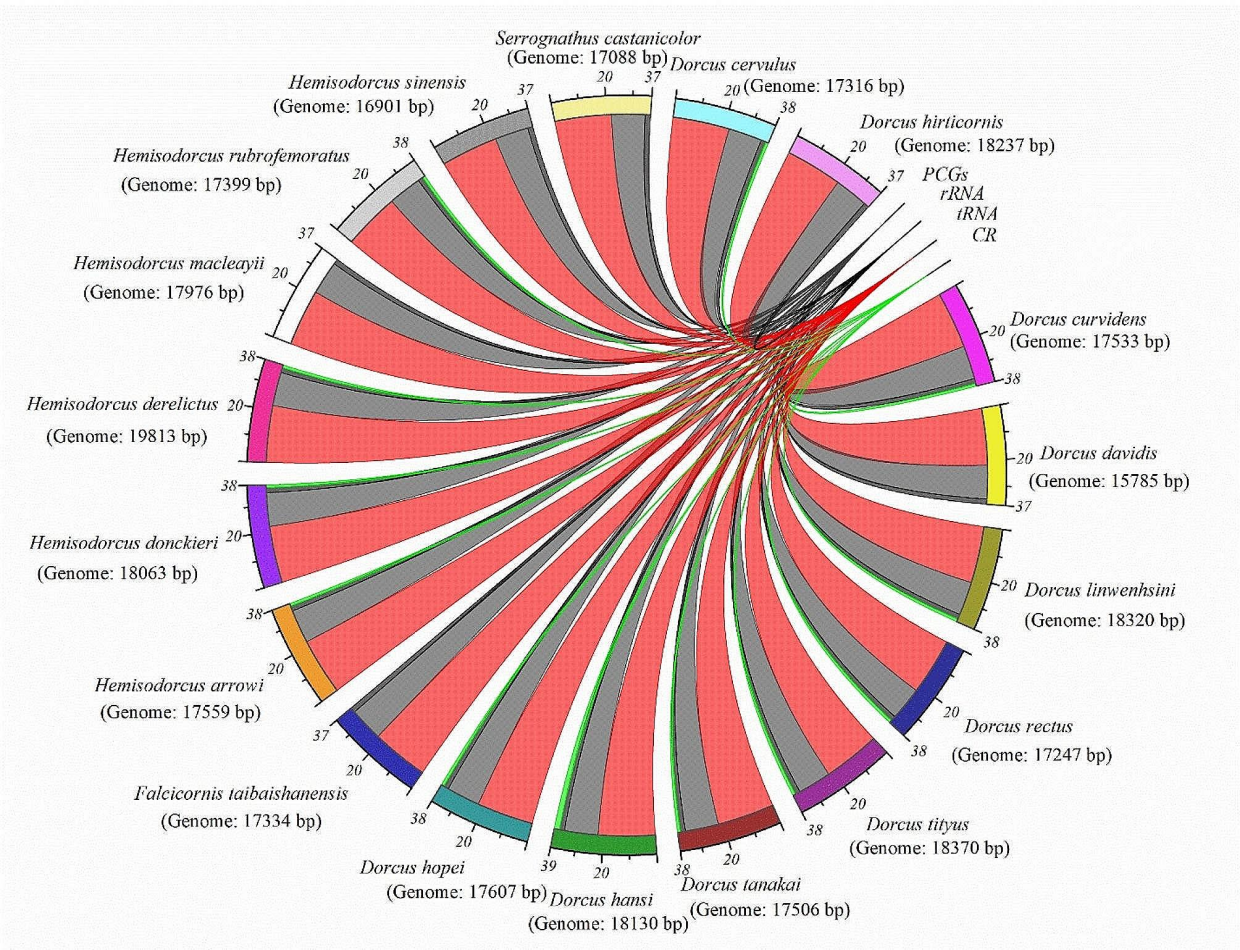
Genome composition of 18 newly identified Eastern Asian *Dorcus* species from China. (Note: The data has been extracted from the annotated genomes and Chord-diagram has been constructed through Origin Pro 2022 software)

In current study, mitochondrial genomes of 18 *Dorcus* s.l. specimens (*D. curvidens, D. davidis, D. linwenhsini, D. rectus, D. tityus, D. tanakai, D. hansi, D. hopei, F. taibaishanensis, H. arrowi, H. donkeiri, H. derelictus, H. macleayii, H. rubrofemoratus, H. sinensis, S. castanicolor, D. cervulus* and *D. hirticornis)* sequenced by Illumina HiSeq 2000 sequencer. The sequencing results represented that all the specimens have circular mitogenomes with the size range 15,785 to 19,813 bp (Fig. 1). Out of all, 12 genomes were composed of 37 genes including 13 PCGs, 2 rRNAs and 22 tRNAs, and a control region (Fig. 1, Fig. S1-Fig. S3). Perhaps due to probability in the practical error, 6 *Dorcus* s.l. (*D. davidis*, *F. taibaishanensis*, *H. macleayii*, *H. sinensis*, *S. castanicolor*, and *D. hirticornis*) gave partially complete mitogenome sequences i.e., sequences contained 37 genes like 13 PCGs, 2 rRNAs, and 22 tRNA and unfortunately didn’t have control region (Fig, S4, Fig. S5). Among these, 23 genes [9 PCGs (*COI, COII, COIII, ATP6, ATP8, NAD2, NAD3, NAD6*, and *Cytb*) and 14 tRNAs *(trnA, trnD, trnE, trnG, trnI, trnK, trnL (uaa), trnM, trnN, trnR, trnS(uga), trnS (ucu), trnT and trnW*)] were present on the J-strand (Also known as major/majority strand) and remaining 14 genes [4 PCGs (*NAD1, NAD4, NAD4L*, and *NAD5*), 2 rRNAs (*rrnl* and *rrns*) and 8 tRNAs (*trnC, trnF, trnH, trnL(uag), trnP, trnQ, trnV*, and *trnY*)] were present on the N-strand (Also known as minus/minority/light strand).

### A + T content and codon usage

The nucleotide compositions of 18 *Dorcus* s.l. mitogenomes have displayed a higher “A + T” content with overall “A + T” contents from 66.29% (*D. linwenhsini*) to 72.85% (*H. donckieri*). Throughout the whole genome, the “AT” skews are positive, but the “GC” skews are negative (Table 1). The data indicates that “A” occurs more frequently than “T”, and “C” occurs more frequently than “G”. In protein-coding regions, there is a negative skew in “AT” values (ranging from − 0.18 to -0.14), and a negative skew in “GC” values (ranging from − 0.07 to -0.03) across all species. This means that there is a bias towards “T” and “C” in protein-coding regions. When analyzing tRNA, there is a strong preference for “G” over “C” (with GC skews ranging from 0.09 to 0.12), and a preference for “A” over “T” (with AT skews ranging from 0.02 to 0.05). Lastly, the rRNA genes demonstrate negative AT-skew values (ranging from − 0.12 to -0.07) and positive GC-skew values (ranging from 0.37 to 0.41), indicating a significant preference for “T” and “G”. This information is summarized in Table 1.

**Table 1:** AT-content, AT-skew, and GC-skew of 18 *Dorcus* s.l. mitochondrial genomes

Dorcus species	Genome			PCGs			tRNAs			rRNAs		
	A + T%	AT-skew	GC-skew	A + T%	AT-skew	GC-skew	A + T%	AT-skew	GC-skew	A + T%	AT-skew	GC-skew
*Dorcus curvidens*	69.14	0.06	-0.32	67.70	-0.16	-0.06	72.04	0.04	0.11	72.32	-0.11	0.41
*Dorcus davidis*	71.25	0.05	-0.27	70.57	-0.16	-0.04	74.59	0.03	0.11	75.95	-0.07	0.39
*Dorcus linwenhsini*	66.29	0.05	-0.33	66.87	-0.15	-0.07	71.64	0.04	0.10	71.73	-0.09	0.39
*Dorcus rectus*	68.90	0.05	-0.28	66.90	-0.15	-0.06	72.61	0.03	0.11	73.26	-0.09	0.38
*Dorcus tityus*	71.37	0.03	-0.29	69.69	-0.18	-0.04	72.92	0.03	0.12	75.07	-0.08	0.39
*Dorcus tanakai*	70.34	0.04	-0.29	69.32	-0.16	-0.05	73.35	0.04	0.11	73.58	-0.08	0.40
*Dorcus hansi*	70.38	0.06	-0.32	68.28	-0.16	-0.04	73.35	0.05	0.09	73.03	-0.07	0.38
*Dorcus hopei*	68.58	0.08	-0.31	66.96	-0.15	-0.06	71.70	0.03	0.11	72.12	-0.10	0.39
*Falcicornis taibaishanensis*	70.12	0.06	-0.28	69.60	-0.15	-0.04	73.44	0.04	0.10	73.48	-0.08	0.39
*Hemisodorcus arrowi*	70.63	0.04	-0.29	69.08	-0.16	-0.04	72.77	0.03	0.10	74.52	-0.09	0.38
*Hemisodorcus donckieri*	72.85	0.04	-0.32	70.81	-0.16	-0.05	75.16	0.04	0.12	75.84	-0.08	0.40
*Hemisodorcus derelictus*	71.33	0.04	-0.31	70.27	-0.16	-0.04	73.31	0.03	0.11	75.24	-0.07	0.37
*Hemisodorcus macleayii*	69.54	0.10	-0.33	67.82	-0.14	-0.05	72.69	0.04	0.09	73.12	-0.12	0.40
*Hemisodorcus rubrofemoratus*	70.86	0.05	-0.28	68.67	-0.16	-0.03	72.82	0.04	0.12	74.71	-0.10	0.37
*Hemisodorcus sinensis*	66.83	0.11	-0.28	66.67	-0.16	-0.05	71.48	0.03	0.12	72.64	-0.11	0.37
*Dorcus cervulus*	68.73	0.05	-0.31	67.25	-0.15	-0.07	72.64	0.02	0.11	72.98	-0.07	0.38
*Dorcus hirticornis*	68.19	0.06	-0.34	66.61	-0.15	-0.07	71,81	0.02	0.12	72.25	-0.09	0.41
*Serrognathus castanicolor*	69.19	0.07	-0.28	67.56	-0.16	-0.07	73.70	0.05	0.09	73.10	-0.11	0.37

**Fig. 2:**
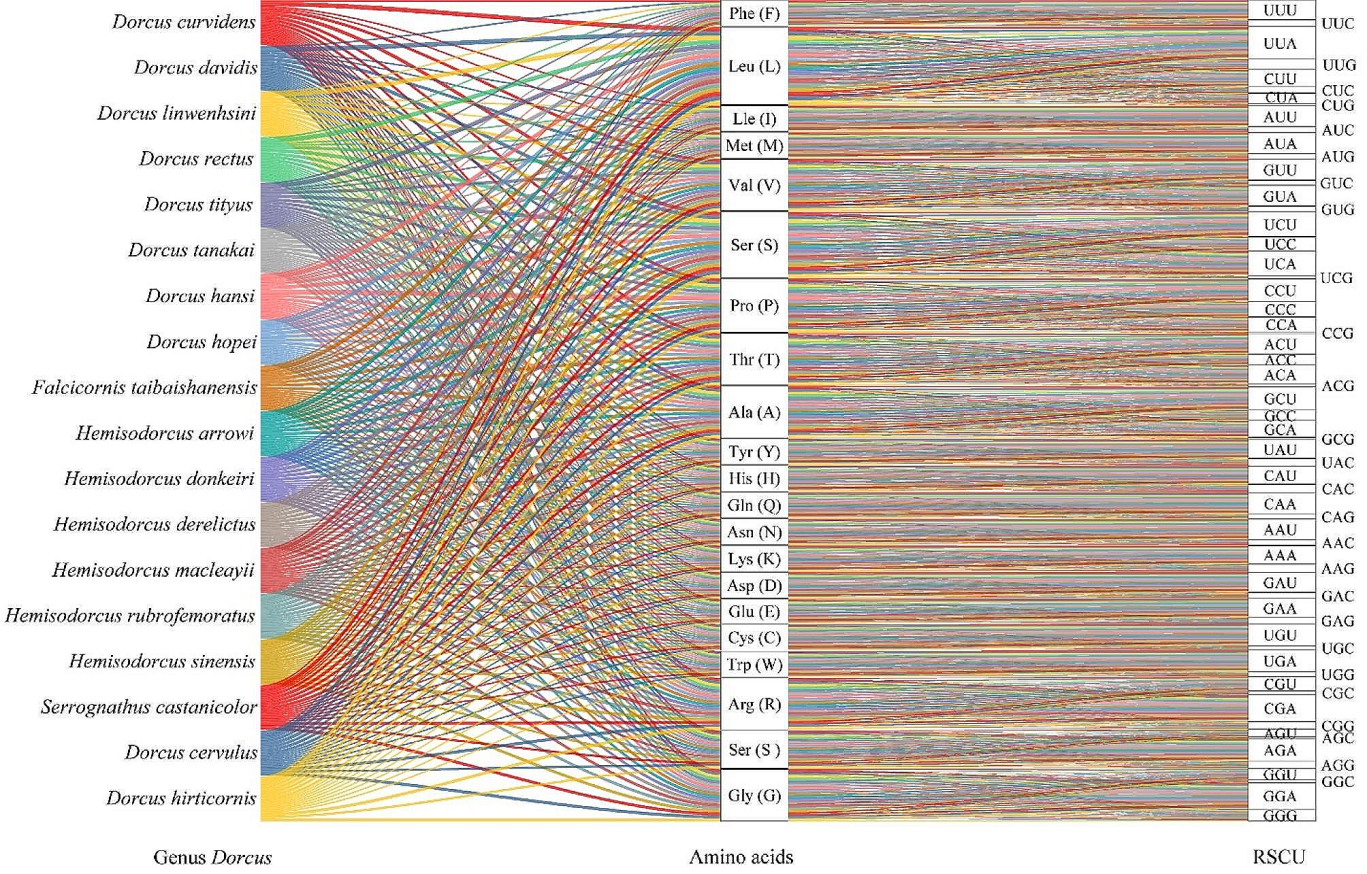
The relative synonymous codon usage (RSCU) of the 18 Eastern Asian *Dorcus* stage beetles mitogenomes. Note: The strength of the thread indicates the RSCU value

The 12 PCGs in 18 *Dorcus* s.l. mitogenomes are initiated with the standard start codon “ATN” (“ATA”, “ATG”, “ATC” and “ATT”), and the start codon of *COI* is “AAT” or “AAC” (Table S1). All PCGs in 18 newly sequenced mitogenomes of *Dorcus* s.l. terminated with “TAA”, “TAG”, “TA”, or “T” codons. Among the PCGs, *COII, COIII, ND4* and *ND5* in majority of the *Dorcus* s.l. terminated with incomplete stop codons (Table S2). The codons “ATT” (Iie (Isoleucine)), “TTA” (Leu (Leucine)), “TTT” (Phe (Phenylalanine)), and “ATA” (Met (Methionine)) are the four most commonly used codons in the mitotic genome of *Dorcus* s.l. in Table S1 and Table S2. The relative synonymous codon usage (RSCU) patterns of these 18 *Dorcus* are roughly the same, with RSCU values shown in Fig. 2.

### Evolutionary rates of PCGs

**Fig. 3:**
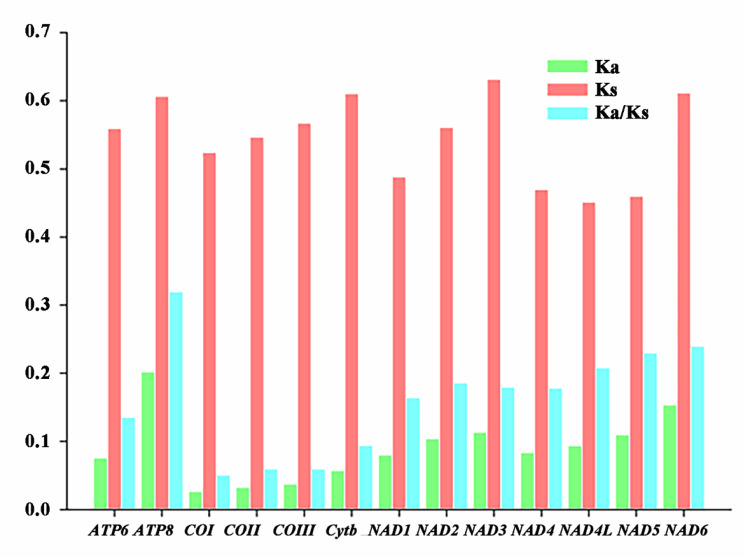
Evolution rate of mitochondrial protein-coding genes of newly identified 18 Eastern Asian *Dorcus* stag beetles

The evolutionary rates of PCGs were examined in all newly sequenced mitogenomes of *Dorcus* s.l. The ratio of nonsynonymous substitutions (Ka) to synonymous substitutions (Ks) were calculated for each PCG (Fig. 3). The Ka/Ks value of the 13PCGs among all new mitogenomes of *Dorcus* s.l. is less than 1.0, indicating that they are all under strong purifying selection. This means, synonymous substitutions occurs at a faster rate than the nonsynonymous substitutions (under strongest selection pressure). The cytochrome oxidase subunits (*COI, COII*, and *COIII*) and cytochrome b (*Cytb*) exhibited lower Ka/Ks ratios compared to ATP synthase subunits (*ATP8* and *ATP6*) and NADH dehydrogenase subunits (*ND1-6* and *4 L*). The order of Ka/Ks of 13 PCGs is *ATP8 > NAD6 > NAD5 > NAD4L > NAD2 > NAD3 > NAD4 > NAD1 > ATP6 > Cytb > COIII > COII > COI*. The fastest evolutionary rate was observed in *ATP8* while the slowest rate was noted *COI* gene in all *Dorcus* mitogenomes (Fig. 3).

### Intergenic spacers

**Fig. 4:**
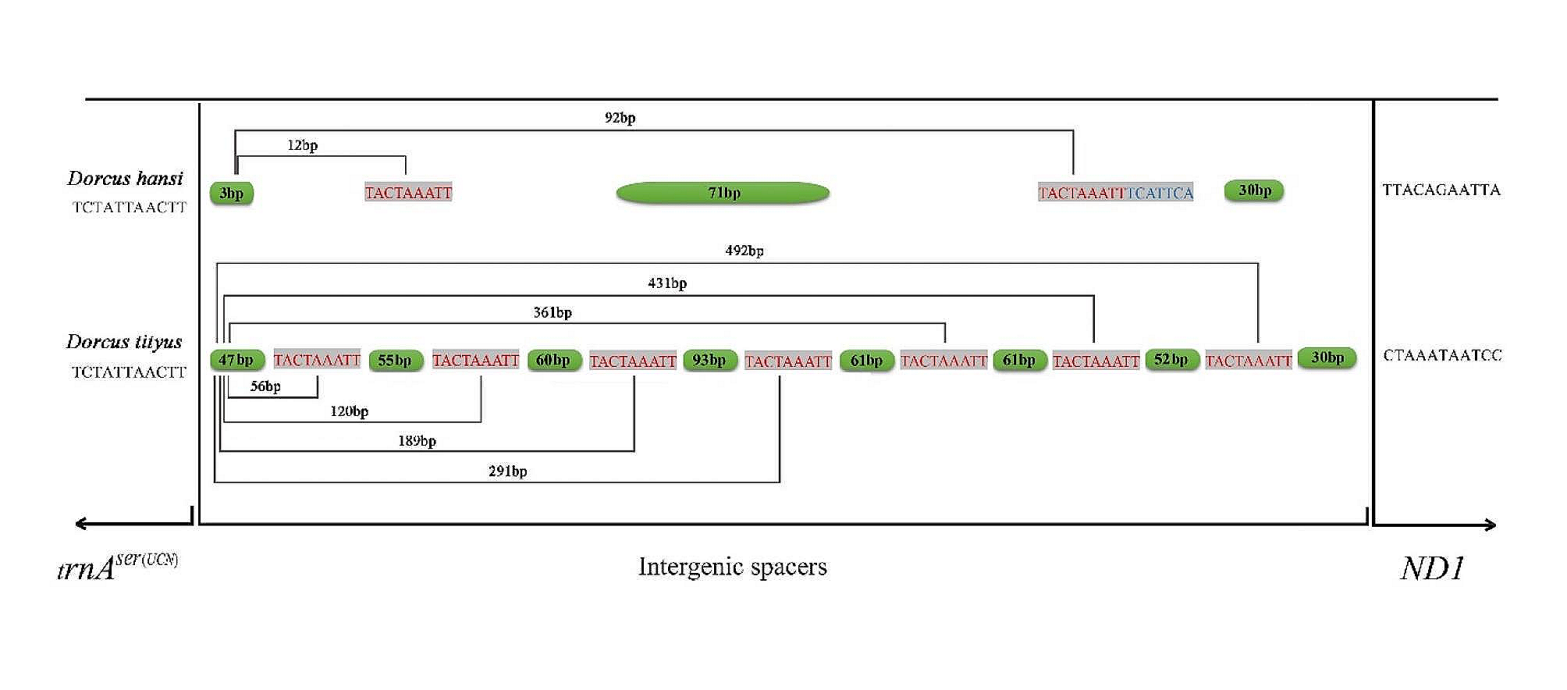
Composition of the large intergenic spacer between trnAser(UCN) and *NAD1* among the two mitochondrial genomes in the present study. The light grey-shaded region is the short sequence repeat (TACTAAATT). The light green-shaded region indicates the length of the spacers between the short sequence repeats

Among the 18 newly sequenced mitogenomes, large intergenic spacers (IGS) are only found in *Dorcus hansi* and *Dorcus tityus*. IGSs more than 30 bp are only observed between *trnA*^*ser(UCN)*^ and *NAD1*. A short sequence (TACTAAATT) repeatedly occurred in large IGSs, while the locations and time of repetition have variability in its existence in *D. hansi* and *D. tityus.* Comparison of *D. hansi* and *D. tityus* (Fig. 4) reveals that the IGS of *D. hansi* is 129 bp with two discontinuous short sequences (TACTAAATT), while the IGS of *D. tityus* is 532 bp and there are seven discontinuous short sequences (TACTAAATT) in this sequence, with 47 bp–, 55 bp–, 60 bp–, 93 bp–, 61 bp–, 61 bp–, 52 bp–, 40 bp– long intergenic region among of the seven repeats from the 5´ to 3´, respectively (Fig. 4).

### Phylogenetic relationships

**Fig. 5:**
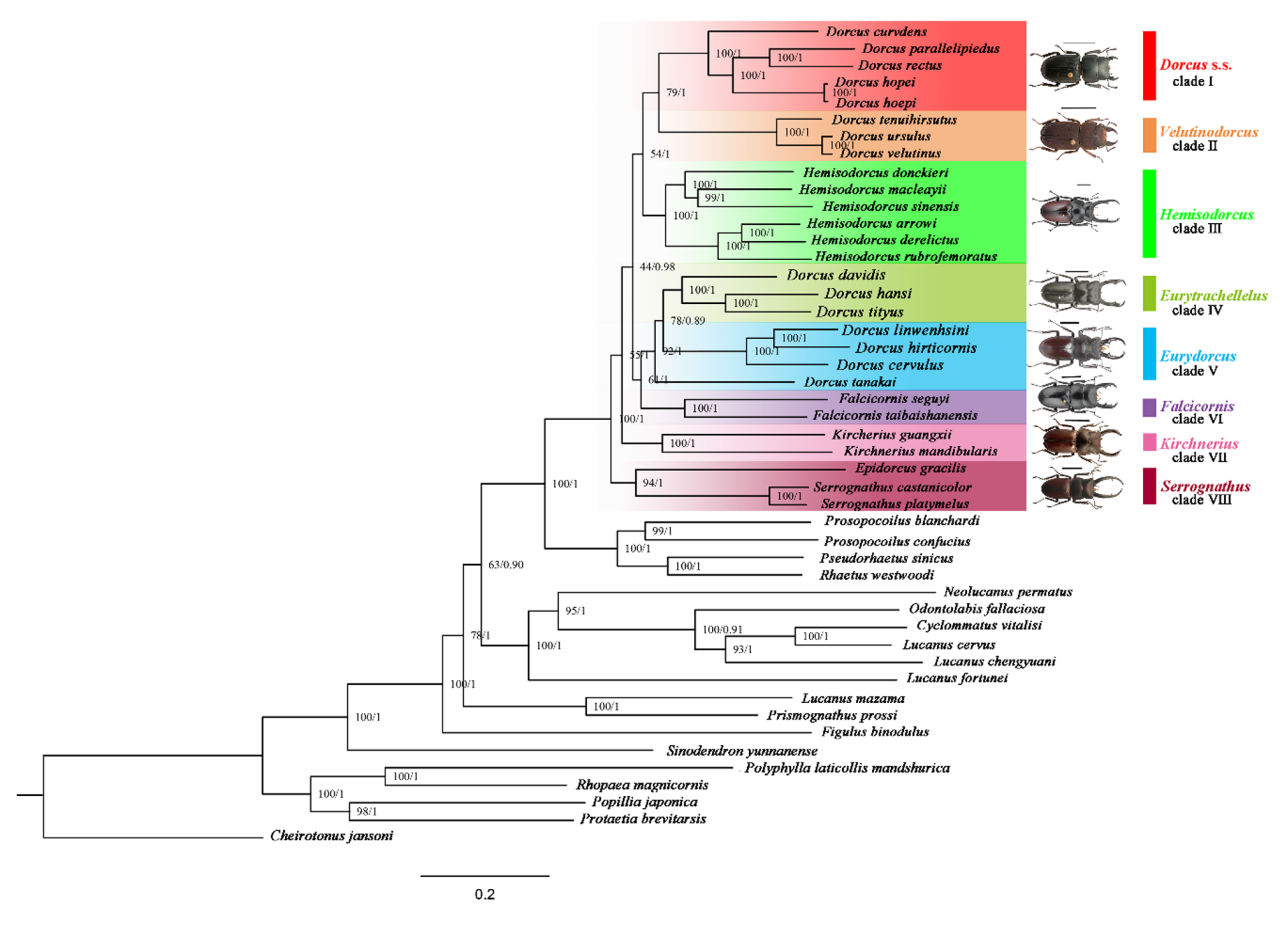
Phylogenetic reconstruction of East Asian *Dorcus* Stag Beetles: Integrating maximum likelihood method and Bayesian inferences with 13 protein-coding genes (PCGs) and 2 rRNAs.

Including newly sequenced 18 *Dorcus* s.l. mitogenomes, a total of 42 Lucanidae mitogenomes as ingroups and five Scarabaeidea genomes as outgroups considered for reconstruction of phylogenetic tree based on different genomic datasets like 13 PCGs and 13 PCGs + 2 rRNAs (*rrnl* and *rrns*). Phylogenetic analysis was performed with “Maximum Likelihood” and “Bayesian Inferences”. The trees constructed with IQtree and PhyloBayes (Fig. 5, Fig. S6) have similar topologies for two data sets, thereby strongly supporting the monophyly of *Dorcus* and formed a sister group relationship with *Prosopocoilus* and *Rhaetus*. The representative species in this genus are clustered into the following eight clades (Fig. 5, Fig. S6). Clade VIII is the *Serrognathus* clade (MLB = 100%, BPP = 1.00), comprising *Serrognathus platymelus*, *Serrognathus castanicolor* and *Epidorcus gracilis*; Clade VII is the *Kirchnerius* clade (MLB = 55%, BPP = 1.00 in Fig. 5; MLB = 47%, BPP = 0.96 in Fig. S6), comprising *Kirchnerius mandibularis* and *Kirchnerius guangxii*; Clade VI is the *Falcicornis* clade (MLB = 64%, BPP = 1.00 in Fig. 5; MLB = 59%, BPP = 1.00 in Fig. S6), comprising *Falcicornis taibaishanensis* and *Falcicornis seguyi*; Clade V is the *Eurydorcus* clade (MLB = 92%, BPP = 1.00 in Fig. 5; MLB = 92%, BPP = 0.98 in Fig. S6), comprising *Dorcus tanakai*, *Dorcus cervulus*, *Dorcus hirticornis* and *Dorcus linwenhsini*; Clade IV is the *Eurytrachellelus* clade (MLB = 78%, BPP = 0.89 in Fig. 5; MLB = 92%, BPP = 0.98 in Fig. S6), comprising *Dorcus tityus*, *Dorcus hansi* and *Dorcus davidis*; Clade III is the *Hemisodorcus* clade (MLB = 54%, BPP = 1.00 in Fig. 5; MLB = 69%, BPP = 0.96 in Fig. S6), comprising *Hemisodorcus rubrofemoratus*, *Hemisodorcus derelictus*, *Hemisodorcus arrowi*, *Hemisodorcus sinensis*, *Hemisodorcus macleayii* and *Hemisodorcus donckieri*; Clade II is the *Velutinodorcus* clade (MLB = 79%, BPP = 1.00 in Fig. 5; MLB = 86%, BPP = 1.00 in Fig. S6), formed by *Dorcus velutinus*, *Dorcus ursulus* and *Dorcus tenuihirsutus*; Clade I is the *Dorcus* s. s. clade (MLB = 79%, BPP = 1.00 in Fig. 5; MLB = 86%, BPP = 1.00 in Fig. S6), formed by *Dorcus hopei*, *Dorcus hopei*, *Dorcus rectus*, *Dorcus parallelipipedus* and *Dorcus curvidens*. Additionally, the genetic distances (K2P-distances) have been calculated among different clades using *COI* genes and significant genetic distance among the different clades has been noticed ranging from 16.1 to 19.6%. The largest genetic distance (19.6%) has been recorded between Clade I and Clade III, Clade V and Clade VIII while the lowest genetic distance (16.1%) has been recorded between Clade III and Clade IV (Table 2).

**Table 2:** The genetic distance for the *Dorcus* stag beetles (Kimura 2-parameter)

	Clade I	Clade II	Clade III	Clade IV	Clade V	Clade VI	Clade VII	Clade VIII	Outgroup
Clade I									
Clade II	0.176								
Clade III	0.174	0.175							
Clade IV	0.173	0.175	0.161						
Clade V	0.191	0.186	0.183	0.175					
Clade VI	0.176	0.179	0.175	0.165	0.180				
Clade VII	0.189	0.184	0.178	0.176	0.190	0.187			
Clade VIII	0.196	0.193	0.187	0.179	0.196	0.189	0.188		
Outgroup	0.211	0.205	0.205	0.204	0.214	0.204	0.211	0.218	

## Discussion

In the current study genome length of all the newly identified *Dorcus* species was in the range of 15,785 to 19,813 bp that are consistent with already identified *Dorcus* species like *D. tenuihirsutus (18,505 bp), D. ursulus (18,820 bp), D. velutinus* complex (14,949 bp without control region i.e., partially complete mitogenomes) [31], and *D. hopei* (16,026 bp) and *D. seguyi* (17,950 bp) [4]. Also, the reported mitogenome size of lucanid members ranges from 15,261 bp (*Lucanus mazama*) to 21,628 bp (*Prosopocoilus astacoides*) [40, 44]. Researchers have suggested that the difference in mitochondrial genome size could be attributed to the variability in tandemly repeated elements within the potential control region, intergenic space, and the repetition of large fragments within both the coding and non-coding regions of the mtDNA [31, 38, 45]. Chen et al. [31] reported that although the genomic length doesn’t have any role in systematics because the overlapping genomic length of beetles belongs to different families and genera the mitochondrial genome of beetles retains the typical gene bases contents and gene organization of the ancestor and the evolutionary rates of all protein-coding genes (PCGs) that denote their evolution is according to purifying selection [31, 46]. All our *Dorcus* genomes have 13 PCG, 2 rRNAs 22 tRNAs, and a control region. Among these, 23 genes (9 PCGs and 14 tRNAs) are present on the majority strand while the minority strand contains the remaining 14 genes. Similar findings have been reported for other Lucanidae species [4, 29–32]. The arrangements of all the newly sequenced *Dorcus* mitogenome share the ancestral type of Lucanidae without rearrangement [4, 30–33].

Although the overall genome of all our *Dorcus* species projected the positive AT skew and negative GC skews for all PCGs, both skews were negative in rRNAs, and in tRNAs, both were positive (Table 1). This trend is recognized as the common ancestral genome character of Lucanidae members [4, 32, 47]. Moreover, the 12 protein-coding genes (PCGs) in these species predominantly initiated with the standard start codons ATN (ATA, ATG, ATC, and ATT), while the *COI* gene specifically started with AAT or AAC as commonly observed in other *Dorcus* genome [4, 31]. Additionally, the PCGs terminated with TAA, TAG, TA, or T codons. Ojala et al. [48] reported that the presence of both complete and incomplete stop codons indicates that post-transcriptional RNA processing mechanisms, such as polyadenylation, may be involved in generating the mature mRNA transcripts. RSCU analysis of different studies revealed the four most frequent used codons [ATT (Ile), TTA (Leu), TTT (Phe), and ATA (Met)] in the *Dorcus* mitogenomes [4, 31, 32, 43]. These codons exhibit a high usage frequency across the 18 mitogenomes, indicating a preference for specific codons during translation. The observation of similar RSCU patterns among the *Dorcus* species suggests a conserved codon usage bias within this genus. Such conservation may be attributed to functional constraints, selective pressures, or shared evolutionary history [31, 32, 40, 49].

The Ka/Ks value of the 13PCGs among all new mitogenomes of *Dorcus* is less than 1.0, indicating that all the PCGs are under strong purifying selection. The evolutionary rates of PCGs in the mitochondrial genome of *Dorcus* showed that their evolution is based upon purifying selection [31, 33, 40, 50]. Additionally, IGS studies have helped to resolve phylogenetic uncertainties, clarify evolutionary lineages, and provide insights into the diversification and biogeography of Lucanidae [27, 36, 40]. The previously reported *Dorcus velutinus* complex also has large IGSs and a short sequence (TACTAAATT), which could provide a unique phylogenetic signal in the genus *Dorcus* [31, 40]. Similarly, *D. tityus* and *D. hansi* also contained large IGS and formed an independent clade with other genera in phylogenetic analysis (Fig. 5, Fig. S6), and the large IGSs once again has played a key role in the classification of *Dorcus*.

This research unveils the first report phylogeny of Eastern Asian *Dorcus* stag beetles. Utilizing two distinct datasets encompassing 13 PCGs and 13 PCGs + rRNA (*rrnl* and *rrns*), the study elucidates robust phylogenetic relationships within Eastern Asian *Dorcus* stag beetles. The overall phylogenetic tree signifies that the taxa included in this study effectively capture the genetic diversity of East Asian *Dorcus* stag beetles, with no apparent impact of long-branch attraction within the ingroup. Both of our methods (ML & BI) illustrated congruent phylogenetic trees. However, the basal relationships, particularly under ML, exhibit less conclusive resolution, potentially attributed to inadequate sampling across diverse lineages. Tree topology in the current study illustrated that *Sinodendron yunnanense* is the earliest branch of Lucanidae while the genus *Figulus* and *Prismognathus* have very close relationships genus Lucanus within the Lucanidae family (Fig. 5) and similar findings have been published by different Biologists [30, 32, 33]. Similarly, tree topology indicated that *Dorcus* s.l. and the genus *Prosopocoilus* have their common ancestors (Fig. 5) which is supported by the finding of Huang who constructed the best-rooted tree by TNT under equal weights with 36 morphometric characters [16]. Alike results have also been documented based on complete mitochondrial genome datasets [30]. Our research divided the *Dorcus* s.l into eight distinct clades. *Serrognathus*, *E. gracilis* was recovered as sister to *S. castanicolor* and *S. platymelus*, this clade was hence named as *Serrognathus*. Saunder (1854) first assigned *E. gracilis* to *Cladognathus* [19], but Van Roon (1910) divided it to the genus *Hemisodorcus* [51]. Séguy (1954) established *Epidorcus* and assigned it as *Epidorcus* [52]. Benesh (1960) then assigned *E. gacilis* to *Prosopocoilus* [5], and Huang & Chen (2013) subsequently assigned *E. gracilis* to *Epidorcus* [3]. However, *E. gracilis* was similar to the typical *Serrognathus* in morphology, most of them were medium to large in size, and the large male had multiple fine small teeth in the upper jaw, while the male phallus valgus sacs in the genitalia were thick and short without bifurcating. *E. gracilis* was distinguished from the typical genus *Prosopocoilus* by the long trifurcation of the male phallus valgus bursa (Fig. S7). Meanwhile, Our result strongly supports the *E. gracilis* sistering to *S. castanicolor* + *S. platymelus* with mitogenomic data, herein, in line with Wan [53, 61]. Within the genus *Kirchnerius* (Fig. S8), *K. guangxii* shares a close affinity with *K. mandibularis* as verified by the comparative mt genome evidence and consistent with Maes classification [54, 55]. *Falcicornis* comprises two species (*F. taibaishanensis* and *F. seguyi*) which are characterised by ventral plate of basal piece triangular and body blackish brown or dark brown colour (Fig. S9). Huang & Chen (2013) divided *D. linwenhsini* into *Dorcus* s. s., however, the current phylogenetic analysis reveals that *D. linwenhsini* is distantly related to *Dorcus* s. s [3].. Most of the body appears flat wide, black or maroon (Fig. S10). The male is characterized by two separate inner teeth in the maxilla, the frontal inner teeth are strongly protruding, and the end teeth of the maxilla are far away from the top of maxilla. Hence, *D. linwenhsini* is classified as *Eurydorcus* with *D. hirticornis, D. tanakai* and *D. cervulus* based on their aforementioned morphological trait similarity. Similarly, *D. tanakai* has resemblance with other members including *D. linwenhsini* and *D. cervulus* with internal teeth of the mandible and maxilla (Fig. S10). According to morphology (Fig. S11) and combined with phylogenetic tree analysis, *D. davidis*, *D. hansi* and *D. tityus* belong to *Eurytrachellelus*. So *Eurydorcus* and *Eurytrachellelus* formed the sister group. *H. donckieri*, *H. macleayii*, *H. sinensis*, *H. arrowi*, *H. derelictus* and *H. rubrofemoratus* clustered into a branch with a high support rate. In addition, their male genital valgus pouches are stronger than those of typical *Dorcus* s. s., and the sac is longer. These features are similar to the genital characteristics of *Hemisodorcus* (Fig. S12). Therefore, these species are assigned to the genus *Hemisodorcus*, which is consistent with the study of Benesh (1960) [5]. The current analysis hinted at the *D. velutinus* complex as being an independent genus of the genus *Dorcus*, as proposed by Chen et al. [31], and the sister group of *Dorcus* s. s. with high node support. In terms of external morphology, the prothorax, back plate and elytra of these three species are extremely rough, covered with brown bristles (Fig. S13) and differ from those of *Dorcus* s. s., therefore, these belonged to the genus *Velutinodorcus*. As the type species of *Dorcus*, *D. parallelipipedus* clustered into a branch with *D. curvidens*, *D. rectus*, *D. hopei* and *D. hopei*, and their node support is highly supported. Morphologically, the upper jaw is not particularly large, with only one large unbranched tooth in the middle (Fig. S14). So *D. curvidens*, *D. rectus*, *D. hopei* and *D. hopei* should be assigned to *Dorcus* s. s. This result supported the studies of Mizunuma and Nagai (1994) [23] and Huang and Chen (2013) [3]. The genetic distances of 13 PCGs and 2 rRNA genes between Lucanidae species were examined to gain further insights into the phylogenetic relationships, and the results are provided in Table S8. The K2P genetic distances of 13 PCGs and 2 rRNA genes between ingroup were all higher than 0.20 (Table S8), thereby further confirming that these genera should be considered as a distinct clade. The Ka/Ks values of the 13PCGs among the 18 *Dorcus* mitogenomes were all less than 1.0, indicating that they are all under strong purifying selection (Table 3; Fig. 2). This finding deepens the existing knowledge on the adaptation of *Dorcus* to the complicated changing environment.

**Table 3:** Collection of *Dorcus* specimens throughout East Mountains of China

MAHU No.	Specimens	Collection site
Do006	*Dorcus curvidens*	China, Guangxi Province, Laibin City, Jinxiu County
Do004	*Dorcus davidis*	China, Anhui Province, Anqing City, Yaoluoping Nature Reserve
He020	*Dorcus linwenhsini*	China, Tibet Autonomous Region, Linzhi City, Bomi County, Yigong
Do005	*Dorcus rectus*	China, Liaoning Province, Shenyang City, Qipan Mountain
Do007	*Dorcus tityus*	China, Tibet Autonomous Region, Linzhi City, Tongmai
Se004	*Dorcus tanakai*	China, Guangxi Province, Laibin City, Jinxiu County
C19	*Dorcus hansi*	China, Guangxi Province, Daming Mountain
Do008	*Dorcus hopei*	China, Guangxi Province, Baise City, Leye County
Ma013	*Falcicornis taibaishanensis*	China, Guangxi Province, Laibai City, Jinxiu County
He014	*Hemisodorcus arrowi*	China, Yunnan Province, Lincang City, Yun County
Do034	*Hemisodorcus donckieri*	China, Tibet Autonomous Region, Linzhi City, Motuo County
He015	*Hemisodorcus derelictus*	China, Tibet Autonomous Region, Linzhi City, Motuo County
Do033	*Hemisodorcus macleayii*	China, Tibet Autonomous Region, Linzhi City, Tongmai Town
He018	*Hemisodorcus rubrofemoratus*	China, Anhui Province, Anqing City, Yaoluoping Nature Reserve
He019	*Hemisodorcus sinensis*	China, Yunnan Province, Lincang City, Yun County
Se002	*Serrognathus castanicolor*	China, Guangxi Province, Laibin City, Jinxiu County
Se008	*Dorcus cervulus*	China, Sichuan Province, Ya’an City
Se006	*Dorcus hirticornis*	China, Yunan Province, Lincang City, Yun County

*Labeling voucher in Museum of Anhui University Hefei, China

**Table 4:** Accession number used for the taxonomic revision of *Dorcus* s.l. species in comparison with other Lucanidae and outgroup

Family	Species	Accession number	References
Lucanidae	*Dorcus curvidens*	OL944342	This study
	*Dorcus davidis*	OL944343	This study
	*Dorcus linwenhsini*	OL944345	This study
	*Dorcus rectus*	OL944346	This study
	*Dorcus tityus*	OL944348	This study
	*Dorcus tanakai*	OL944347	This study
	*Dorcus hansi*	MF621709	This study
	*Dorcus hopei*	OL944344	This study
	*Falcicornis taibaishanensis*	OL944349	This study
	*Hemisodorcus arrowi*	OL944350	This study
	*Hemisodorcus donckieri*	OL944352	This study
	*Hemisodorcus derelictus*	OL944351	This study
	*Hemisodorcus macleayii*	OL944353	This study
	*Hemisodorcus rubrofemoratus*	OL944354	This study
	*Hemisodorcus sinensis*	OL944355	This study
	*Serrognathus castanicolor*	OL944357	This study
	*Dorcus cervulus*	OL944356	This study
	*Dorcus hirticornis*	OL944358	This study
	*Dorcus velutinus*	MK050989	Chen et al. (2020)
	*Dorcus ursulus*	MK050990	Chen et al. (2020)
	*Dorcus tenuihirsutus*	MK050991	Chen et al. (2020)
	*Dorcus hopei*	MF612067	Chen et al. (2018)
	*Dorcus parallelipipedus*	KT876887	Linard et al. (2016)
	*Falcicornis seguyi*	MF612068	Chen et al. (2018)
	*Serrognathus platymelus*	MF612070	Direct submission
	*Kirchnerius mandibularis*	MK134566	Zhou et al. (2020)
	*Epidorcus gracilis*	KP735805	Wu et al. (2015)
	*Prosopocoilus confucius*	KU552119	Lin et al. (2017)
	*Prosopocoilus blanchardi*	KF364622	Kim et al. (2015)
	*Pseudorhaetus sinicus*	KP987575	Wu et al. (2015)
	*Rhaetuswest woodi*	MG159815	Liu et al. (2018)
	*Odontolabis fallaciosa*	MF908524	Wang et al. (2018)
	*Neolucanus permatus*	MF401425	Direct submission
	*Lucanus fortunei*	MF614013	Direct submission
	*Lucanus mazama*	FJ613419	Sheffield et al. (2009)
	*Lucanus cervus*	MN580549	Chen et al. (2019)
	*Lucanus chengyuani*	MK878514	Wang et al. (2019)
	*Prismognathus prossi*	MF614014	Liu et al. (2018)
	*Cyclommatus vitalisi*	MF037205	Liu et al. (2017)
	*Sinodendron yunnanense*	KP735804	Lin et al. (2017)
	*Figulus binodulus*	NC045102	Lee et al. (2019)
	*Kircherius guangxii*	NC048957	Zhai et al. (2020)
Scarabaeidae	*Polyphylla laticollis mandshurica*	KF544959	Kim et al. (2013)
	*Rhopaea magnicornis*	NC013252	Cameron et al. (2009)
	*Popillia japonica*	NC038115	Yang et al. (2018)
	*Protaetia brevitarsis*	NC023453	Kim et al. (2014)
	*Cheirotonus jansoni*	NC023246	Shao et al. (2014)

Moreover, strong genetic evidence supports the notion that species belonging to different clades may also belong to different taxa. Additionally, the K2P distance between different clades provides valuable information for assessing their generic relationships. A study conducted by Wu [58] reported an average inter-genetic K2P distance of 0.220 (range: 0.174–0.259) in Lucanidae. Within closely related lucanid genera, such as *Falcicornis* Planet (1894) and *Dorcus* MacLeay (1819), as well as *Rhaetus* Parry (1864) and *Rhaetulus* Westwood (1871), the K2P distance values were found to be 0.173 and 0.174, respectively [4, 56, 58]. The same difference exists between clades A and B (0.176) of *Cyclommatus*, which are considered different genera [59]. Our study reveals K2P-distance values of 0.161 to 0.196 among different clades, suggesting that these distinct clades could potentially represent different genera.

Conclusively, the current study has provided sufficient information for the identification and classification of 18 newly sequenced *Dorcus* species by mitogenomic information, especially 13 PCGs, rRNAs, and LIGSs. Moreover, phylogenetic analysis based on these genes has classified the genus *Dorcus* into 8 distinct clades/ genera (*Serrognathus* Motschulsky, *Kirchnerius* Schenk, *Falcicornis* Séguy, *Eurydorcus, Eurytrachellelus, Hemisodorcus, Velutinodorcus*, and *Dorcus* s.s. MacLeay). Subsequently, Large IGSs are identified as another key character for the understanding of *Dorcus* systematics especially *D. hansi* and *D. tityus*. Due to the strong purification selection in *Dorcus*, this study could be helpful to enhance our understanding regarding evolution within the genus with the passage of species inclusion from other regions.

## Conclusion

In conclusion, our research successfully unraveled the mitogenomic phylogeny of Eastern Asian *Dorcus* stag beetles (Coleoptera: Lucanidae) and provided a generic taxonomy within the big genus. Through the integration of previously published data with newly sequenced mitochondrial genomes from 42 species, we established the monophyly of *Dorcus* and divided the genus into eight distinct lineages with strong nodal support. Our findings confirm the existence of four recognized genera and reinstate four other genera within *Dorcus*, enhancing our understanding of their evolutionary relationships. Notably, the identification of a unique intergenic spacer (IGS) and specific sequence fragment in *D. tityus* and *D. hansi* offers valuable insights for future phylogenomic reconstruction. The newly generated mitogenomic data provide a valuable resource for further investigations on the ecological and evolutionary aspects of these fascinating beetles, facilitating conservation efforts and sustainable management of their forest habitats.

## Materials and methods

### Samples collection and isolation of genomic DNA

The adult specimens of the genus *Dorus* were collected from the East Mountains of China (Table 3). A total of 18 new *Dorcus* specimens were identified based on their morphological characters [2, 5, 18, 21] and were stored at -20 °C for genetic investigation. All taxa voucher specimens were placed in the museum of Anhui University Hefei, China. For mitogenome investigation, the total genomic DNA was isolated from the muscle tissues of the collected *Dorcus* specimens using DNAeasy Blood & Tissue Kit (Qiagen, Germany). The isolated DNAs were quantified via UV-visible nano-spectrophotometer (Model: Nano-100; ALLSHENG, China) and sequenced. The recently acquired sequence data has been deposited to the database of the National Center for Biotechnology Information (https://www.ncbi.nlm.nih.gov/genbank/). The corresponding accession numbers for these sequences are provided in Table 4.

### PCR amplification and sequencing

The three mitochondrial genes (*COI*, *Cytb*, and 16 S) were used for the amplification of genomic DNA (Table S3). The polymerase chain reactions (PCR) were performed by following the primer’s manufacturer protocol. Briefly, each reaction mixture was prepared in a total volume of 25µL, containing template DNA: 2 µL (with at least 50 ng), 2 × EasyTaqSuperMix (+ dye): 12.5 µL, 1 µM of each primer (forward and reverse): 1 µL, and sterilized double-distilled water (ddH_2_O): 8.5 µL. The PCR amplification was carried out in a thermocycler (Model; company) using the PCR conditions as led temperature: 104 ºC, initial denaturation: 94 ºC/2 min. Subsequently, PCR was run about 35 cycles by following the initial denaturation phase: 94 ºC/40 s, annealing phase: 54–58 ºC/50 s, elongation phase: 70–72 ºC/70 s, and a final extension phase: 72 ºC/7 min. Table S3 provides a list of all primers utilized for DNA amplification. Finally, the amplified PCR products were sequenced using the Illumina HiSeq 2000 platform (Berry Genomics, Neijing, China) using the TruSeq nano DNA Kit [60].

### Sequence assembly, annotation, and composition analysis

We employed IDBA-UD, *a de novo* assembler known for reconstructing longer contigs with high accuracy [61], to assemble high-quality mitogenome reads. It was configured with the K values in the range of 80 to 240 bp. Our approach involved selectively identifying mitogenome assemblies from the assembled contigs by employing BLAST using Sanger sequence data from three anchor loci (*COI, Cytb* & *16 S*) with a minimum similarity threshold of 98%. To assess the accuracy of the mitogenome assemblies, we employed Geneious v6.1.7 (Biomatters Ltd., New Zealand) to map the clean reads back onto the assembled mitogenomes. The mapping was conducted with a tolerance of up to 2% mismatches, 3 bp gap size, and 100 bp minimum overlap. Initial annotations were performed using the invertebrate mitochondrial code on the MITOS Web Server (http://mitos.bioinf.uni-leipzig.de/index.py). Protein-coding genes were identified by aligning them with previously published genome sequences in Geneious v6.1.7. Moreover, the rRNAs (*rrnl* and *rrns*) were computed based on sequence similarity with closely related species [62]. Additionally, to gain insights into the genome composition, we determined nucleotide composition, codon usage, and relative synonymous codon usage (RSCU) using MEGA-X [63]. Subsequently, composition skew analysis was performed using different formulas such as AT skew = [A-T]/[A + T] and GC skew = [G-C]/[G + C] [32]. Finally, we calculated the evolutionary rates (Ka/Ks ratios) for each protein-coding gene using DnaSP v5.0 [64].

### Computation of Dorcus phylogeny and genetic distance

Phylogenetic analyses of 18 newly sequenced *Dorcus* mitogenomes were carried out along with 24 Lucanidae mitogenomes available in the GenBank as ingroups (Table 3). Five mitochondrial genomes from Scarabaeidae genomes were also retrieved from the GenBank for the outgroup (Table 3). Individually, we extracted the sequences of each coding gene from the annotated genomes using Geneious Prime v2019.1.1 and aligned using the MAFFT v7.263 [65, 66]. Gaps and sites of undefined alignment were filtered from the data using Gblocks v0.91b [67]. Phylogenetic analyses were assembled based on 2 datasets of the mitochondrial genome: [1] the “PCG matrix” (including 13 PCGs); [2] the “PCGR matrix” (including 13 PCGs and 2rRNA). The selection of the optimal model for each dataset was performed using PartitionFinder 2 in Geneious Prime [68]. An input configuration file was generated, which included 37 predefined partitions based on genes. Unlinked branch lengths and a greedy search algorithm were employed to estimate the best-fitting schemes, while the Akaike Information Criterion (AIC) was used to search for the most suitable scheme (76). Two distinct algorithms, maximum likelihood (ML) and Bayesian inference (BI), were employed for conducting phylogenetic analyses.

Maximum likelihood analysis was performed by uploading a splicing file to the IQ-TREE Web Server (IQ-TREE: Efficient phylogenomic software by maximum likelihood (iqtree.org)). Set the “automatic” option under the optimal evolutionary model and build a phylogenetic tree using an ultra-fast bootstrapping approximation method with 10,000 replicates using SH-aLRT branch test, 0.5 perturbation strength and IQ-TREE stopping rule set as 100 in IQ-TREE search parameters [69]. BI analysis was performed using MrBayes 3.2.6 [70] and a selected site-heterogeneous mix model (GTR + CAT) [30]. Two independent chains started with a random tree and simulated 20,000 generations, where the tree was sampled every 10 generations. Each Markov chain Monte Carlo (MCMC) run in which the first 25% of the tree is excluded as aging. To achieve consensus, a total of 1500 trees obtained from both runs were combined, ensuring that the two runs converged with a maximum difference (maxdiff) below 0.1. to visualize and root the phylogenetic trees, Figtree v1.4.4 [31] was utilized, with the five species in Scarabaeidae serving as outgroups. Moreover, we estimated the average genetic distance among different lineages of taxa using MEGA 11 via K2P distance.

### Electronic supplementary material

Below is the link to the electronic supplementary material.

Supplementary Material 1

## Data Availability

The datasets generated and/or analyzed during the current study are included in this published article [and its supplementary information files].
