# Supplementary material for: The first mitogenomic phylogenetic framework of Dorcus sensu lato (Coleoptera: Lucanidae), with an emphasis on generic taxonomy in Eastern Asia
Source: BMC Ecol Evol. 2024 May 21;24:66. doi: 10.1186/s12862-024-02225-2 (PMC11107052; doi:10.1186/s12862-024-02225-2)
Supplement: Supplementary file 1 — Supplementary Material 1 [file 12862_2024_2225_MOESM1_ESM.docx]

**Unraveling the Mitogenomic Phylogeny of** **Eastern Asian *Dorcus* Stag Beetles (Coleoptera: Lucanidae): Implications for the Taxonomic Revision of the Genus**

**Muhammad Jafir:** *Department of Ecology, School of Resources and Environmental Engineering, Anhui University, Hefei 230601, Anhui, China*. (m.jafir@ahu.edu.cn)

**Liyang Zhou:** *Department of Ecology, School of Resources and Environmental Engineering, Anhui University, Hefei 230601, Anhui, China*. (zhouly1102@163.com)

**Yongjing Chen:** *Department of Ecology, School of Resources and Environmental Engineering, Anhui University, Hefei 230601, Anhui, China.* (1452394259@qq.com)

**Mengqiong Xu:** *Department of Ecology, School of Resources and Environmental Engineering, Anhui University, Hefei 230601, Anhui, China.* (1549142548@qq.com)

**Xia Wan:** *Department of Ecology, School of Resources and Environmental Engineering, Anhui University, Hefei 230601, Anhui, China*. (wanxia@ahu.edu.cn)

***Corresponding author:** wanxia@ahu.edu.cn (Xia Wan)

Links for the studied taxa genome data are available at NCBI database.

1. [Dorcus curvidens mitochondrion, complete genome - Nucleotide - NCBI (nih.gov)](https://www.ncbi.nlm.nih.gov/nuccore/OL944342)
2. [Dorcus davidis mitochondrion, complete genome - Nucleotide - NCBI (nih.gov)](https://www.ncbi.nlm.nih.gov/nuccore/OL944343)
3. [Dorcus linwenhsini mitochondrion, complete genome - Nucleotide - NCBI (nih.gov)](https://www.ncbi.nlm.nih.gov/nuccore/OL944345)
4. [Dorcus rectus mitochondrion, complete genome - Nucleotide - NCBI (nih.gov)](https://www.ncbi.nlm.nih.gov/nuccore/OL944346)
5. [Dorcus tityus mitochondrion, complete genome - Nucleotide - NCBI (nih.gov)](https://www.ncbi.nlm.nih.gov/nuccore/OL944348)
6. [Dorcus tanakai mitochondrion, complete genome - Nucleotide - NCBI (nih.gov)](https://www.ncbi.nlm.nih.gov/nuccore/OL944347)
7. [Dorcus hansi mitochondrion, complete genome - Nucleotide - NCBI (nih.gov)](https://www.ncbi.nlm.nih.gov/nuccore/MF621709)
8. [Dorcus hopei mitochondrion, complete genome - Nucleotide - NCBI (nih.gov)](https://www.ncbi.nlm.nih.gov/nuccore/OL944344)
9. [Falcicornis taibaishanensis mitochondrion, complete genome - Nucleotide - NCBI (nih.gov)](https://www.ncbi.nlm.nih.gov/nuccore/OL944349)
10. [Hemisodorcus arrowi mitochondrion, complete genome - Nucleotide - NCBI (nih.gov)](https://www.ncbi.nlm.nih.gov/nuccore/OL944350)
11. [Hemisodorcus donckieri mitochondrion, complete genome - Nucleotide - NCBI (nih.gov)](https://www.ncbi.nlm.nih.gov/nuccore/OL944352)
12. [Hemisodorcus derelictus mitochondrion, complete genome - Nucleotide - NCBI (nih.gov)](https://www.ncbi.nlm.nih.gov/nuccore/OL944351)
13. [Hemisodorcus macleayii mitochondrion, complete genome - Nucleotide - NCBI (nih.gov)](https://www.ncbi.nlm.nih.gov/nuccore/OL944353)
14. [Hemisodorcus rubrofemoratus mitochondrion, complete genome - Nucleotide - NCBI (nih.gov)](https://www.ncbi.nlm.nih.gov/nuccore/OL944354)
15. [Hemisodorcus sinensis mitochondrion, complete genome - Nucleotide - NCBI (nih.gov)](https://www.ncbi.nlm.nih.gov/nuccore/OL944355)
16. [Serrognathus castanicolor mitochondrion, complete genome - Nucleotide - NCBI (nih.gov)](https://www.ncbi.nlm.nih.gov/nuccore/OL944357)
17. [Dorcus cervulus mitochondrion, complete genome - Nucleotide - NCBI (nih.gov)](https://www.ncbi.nlm.nih.gov/nuccore/OL944356)
18. [Dorcus hirticornis mitochondrion, complete genome - Nucleotide - NCBI (nih.gov)](https://www.ncbi.nlm.nih.gov/nuccore/OL944358)

Table S1. Start codon usage of 13 protein-coding genes of 18 newly sequenced *Dorcus* s.l.

| Species  Gene | *ND2* | *COI* | *COII* | *ATP8* | *ATP6* | *COIII* | *ND3* | *ND5* | *ND4* | *ND4L* | *ND6* | *Cytb* | *ND1* |
| --- | --- | --- | --- | --- | --- | --- | --- | --- | --- | --- | --- | --- | --- |
| *Dorcus curvidens* | ATA | AAC | ATT | ATT | ATA | ATG | ATA | ATA | ATA | ATG | ATG | ATG | ATA |
| *Dorcus davidis* | ATT | AAT | ATT | ATT | ATG | ATG | ATA | ATA | ATG | ATG | ATA | ATG | ATA |
| *Dorcus linwenhsini* | ATA | AAC | ATT | ATT | ATA | ATG | ATA | ATA | ATA | ATG | ATG | ATG | ATA |
| *Dorcus rectus* | ATA | AAC | ATT | ATT | ATA | ATG | ATA | ATA | ATA | ATG | ATG | ATG | ATA |
| *Dorcus tityus* | ATA | AAT | ATT | ATT | ATA | ATG | ATA | ATA | ATA | ATG | ATG | ATG | ATA |
| *Dorcus tanakai* | ATT | AAT | ATT | ATT | ATG | ATG | ATA | ATA | ATG | ATG | ATA | ATG | ATA |
| *Dorcus hansi* | ATA | AAT | ATT | ATT | ATA | ATG | ATA | ATA | ATG | ATG | ATG | ATG | ATA |
| *Dorcus hopei* | ATA | AAC | ATT | ATT | ATA | ATG | ATA | ATA | ATA | ATG | ATG | ATG | ATA |
| *Falcicornis taibaishanensis* | ATA | AAC | ATT | ATC | ATA | ATG | ATA | ATA | ATA | ATG | ATG | ATG | ATA |
| *Hemisodorcus arrowi* | ATA | AAC | ATT | ATT | ATG | ATG | ATT | ATA | ATG | ATG | ATA | ATG | ATA |
| *Hemisodorcus donckieri* | ATA | AAT | ATT | ATT | ATA | ATG | ATA | ATA | ATA | ATG | ATG | ATG | ATA |
| *Hemisodorcus derelictus* | ATA | AAC | ATT | ATT | ATG | ATG | ATT | ATA | ATG | ATG | ATA | ATG | ATA |
| *Hemisodorcus macleayii* | ATA | AAT | ATT | ATT | ATA | ATG | ATA | ATA | ATA | ATG | ATA | ATG | ATA |
| *Hemisodorcus rubrofemoratus* | ATA | AAC | ATT | ATT | ATA | ATG | ATA | ATA | ATA | ATG | ATG | ATG | ATA |
| *Hemisodorcus sinensis* | ATA | AAT | ATT | ATA | ATA | ATG | ATA | ATG | ATA | ATG | ATG | ATG | ATA |
| *Serrognathus castanicolor* | ATT | AAC | ATT | ATT | ATG | ATG | ATT | ATA | ATG | ATG | ATA | ATG | ATA |
| *Dorcus cervulus* | ATT | AAC | ATT | ATT | ATG | ATG | ATT | ATA | ATG | ATG | ATA | ATG | ATA |
| *Dorcus hirticornis* | ATA | AAC | ATT | ATT | ATG | ATG | ATT | ATG | ATG | ATG | ATA | ATG | ATA |

Table S2. Stop codon usage of 13 protein coding genes of 18 newly sequenced *Dorcus* s.l. species.

| Species  Gene | *ND2* | *COI* | *COII* | *ATP8* | *ATP6* | *COIII* | *ND3* | *ND5* | *ND4* | *ND4L* | *ND6* | *Cytb* | *ND1* |
| --- | --- | --- | --- | --- | --- | --- | --- | --- | --- | --- | --- | --- | --- |
| *Dorcus curvidens* | TAA | TAA | T | TAA | TAA | TA | TAG | TA | TA | TAA | TAA | TAG | TAG |
| *Dorcus davidis* | TAA | TAA | T | TAG | TAA | TA | TAG | TA | TA | TAA | TAA | TAG | TAG |
| *Dorcus linwenhsini* | TAA | TAA | T | TAG | TA | TA | TA | T | T | TAA | TAA | TAG | TAG |
| *Dorcus rectus* | TAA | T | T | TAG | TAA | TA | TAG | T | TA | TAA | TAA | TAG | TAG |
| *Dorcus tityus* | TAA | TAA | T | TAA | TAA | TA | TAG | T | TA | TA | TAA | TAG | TAG |
| *Dorcus tanakai* | TAA | TAA | T | TAG | TAA | TA | TAG | TA | TA | TAA | TAA | TAG | TAG |
| *Dorcus hansi* | TAA | TAA | T | TAA | T | TA | T | T | T | TAA | TAA | TAG | TAA |
| *Dorcus hopei* | TAA | TAA | T | TAA | TAA | TA | TAG | TA | TA | TAA | TAA | TAG | TAG |
| *Falcicornis taibaishanensis* | TAA | TAA | T | TAA | TAA | TA | TAG | T | TA | TAA | TAA | TAG | TAG |
| *Hemisodorcus arrowi* | TAA | TAA | T | TAG | TAA | TA | TAG | T | TA | TAA | TAA | TAG | TAG |
| *Hemisodorcus donckieri* | TAA | TAA | T | TAG | TAA | TA | TAG | T | TA | TAA | TAA | TAG | TAG |
| *Hemisodorcus derelictus* | TAA | TAA | T | TAG | TAA | TA | TAG | T | TA | TAA | TAA | TAG | TAG |
| *Hemisodorcus macleayii* | T | T | T | TAG | TAA | TA | TAG | T | TAG | TAA | TAA | TAG | TAG |
| *Hemisodocus rubrofemoratus* | TAA | TAA | T | TAG | TA | TA | TA | T | TA | TAA | TAA | TAG | TAG |
| *Hemisodorcus sinensis* | TAA | TAA | T | TAG | TA | TA | TA | T | TA | TAA | TAA | TAG | TAG |
| *Serrognathus castanicolor* | TAA | TAA | T | TAG | TAA | TA | TAG | T | TA | TAA | TAA | TAG | TAG |
| *Dorcus cervulus* | TAA | TAA | T | TAA | TAA | TA | TAG | T | T | TAA | TAA | TAG | TAG |
| *Dorcus hirticornis* | TAA | TAA | T | TAA | TAA | TA | TAG | TA | TA | TAA | TAA | TAG | TAG |

Table S3. Mitochondrial genes primers used for the amplification of isolated DNA of *Dorcus* s.l. specimens.

| Gene | Primer Name | Primer’s sequence (5´-3´) | References |
| --- | --- | --- | --- |
| Cytochrome c oxidase subunit I (COI) | COI-F | CAACATTTATTTTGATTTTTTGG | (1) |
|  | COI-R | TCCAATGCACTAATCTGCCATATTA |  |
| Cytochrome b | Cytb-F | GAGGAGCAACTGTAATTACTAA | (2) |
|  | Cytb-R | AAAAGAAARTATCATTCAGGTTGAAT |  |
| 16SrDNA | 16S-F | CCGGTTTGAACTCAGATCATG | (3) |
|  | 16S-R | TAATTTATTGTACCTTGTGTATCAG |  |


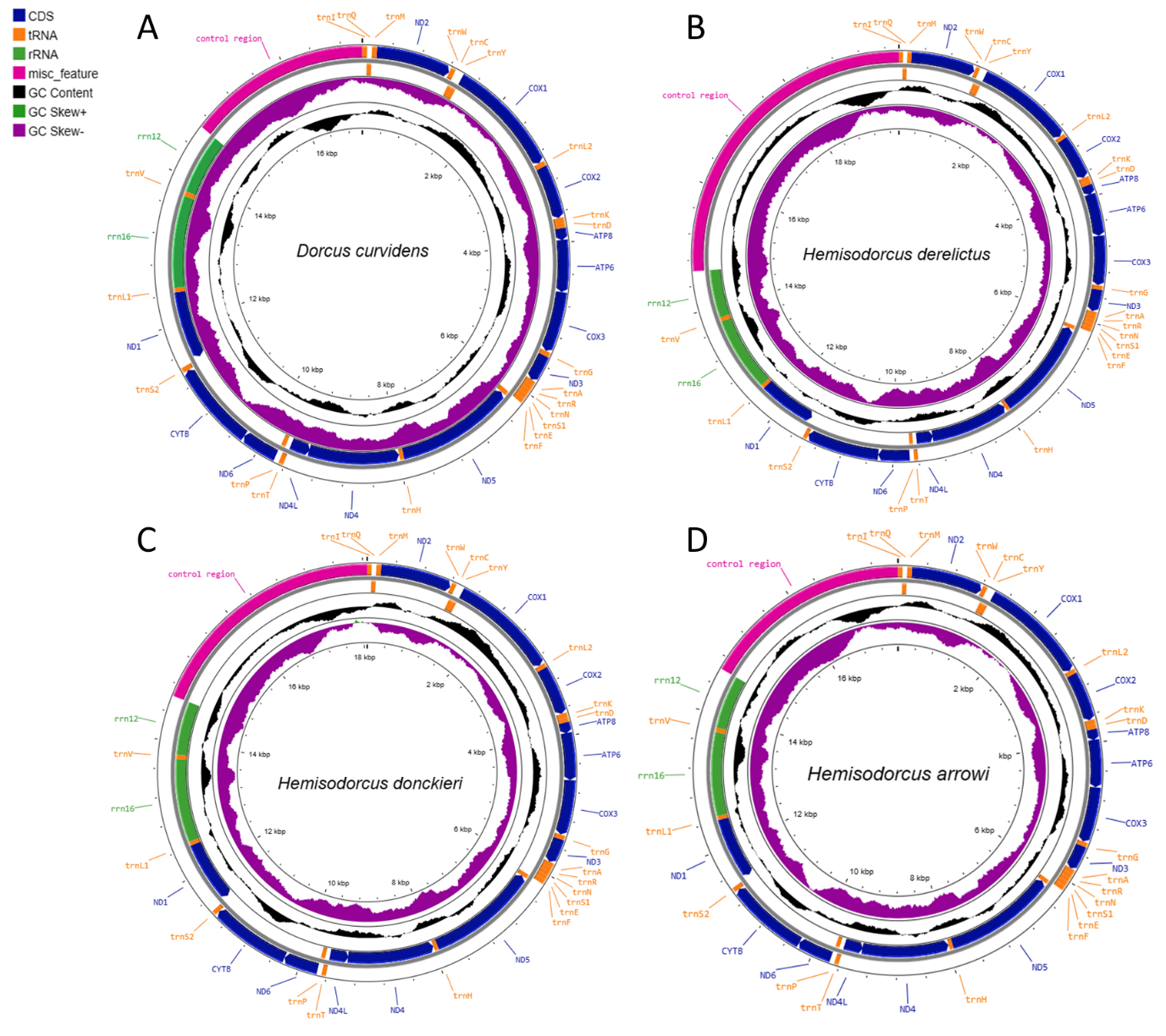


Fig. S1. Completely sequenced mitochondrial genome of *Dorcus* specimens including all the genes and control region. A: *Dorcus* *curvidens* (Accession No. OL944342), B: *Hemisodorcus* *derelictus* (Accession No. OL944351), C: *Hemisodorcus donkieri* (Accession No. OL944352), D: *Hemisodorcus arrowi* (Accession No. OL944350).


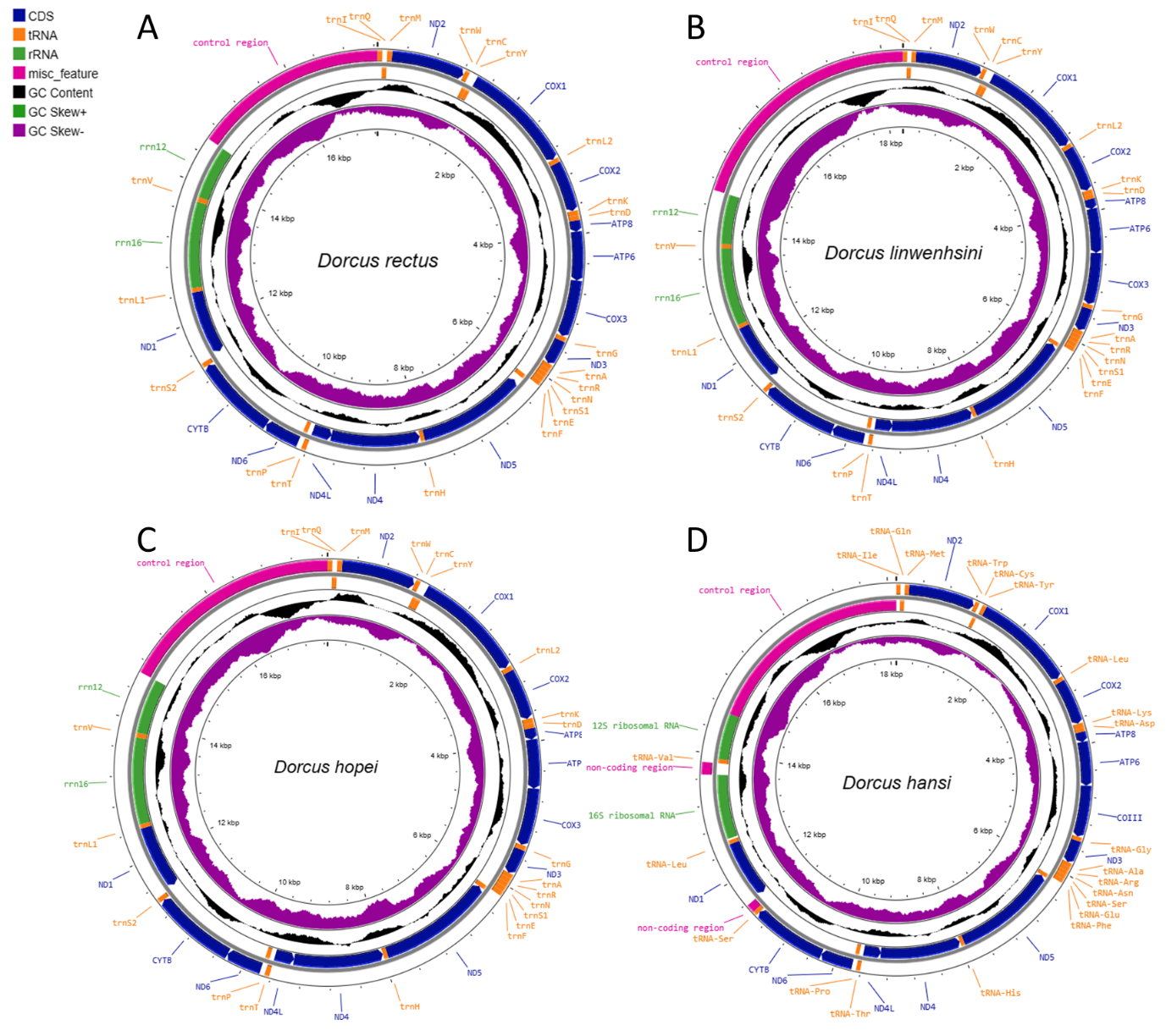


Fig. S2. Completely sequenced mitochondrial genome of *Dorcus* specimens including all the genes and control region. A: *Dorcus* *rectus* (Accession No. OL944346), B: *Dorcus* *linwenhsini* (Accession No. OL944345), C: *Dorcus hopei* (Accession No. OL944344), D: *Dorcus hansi* (Accession No. MF621709).


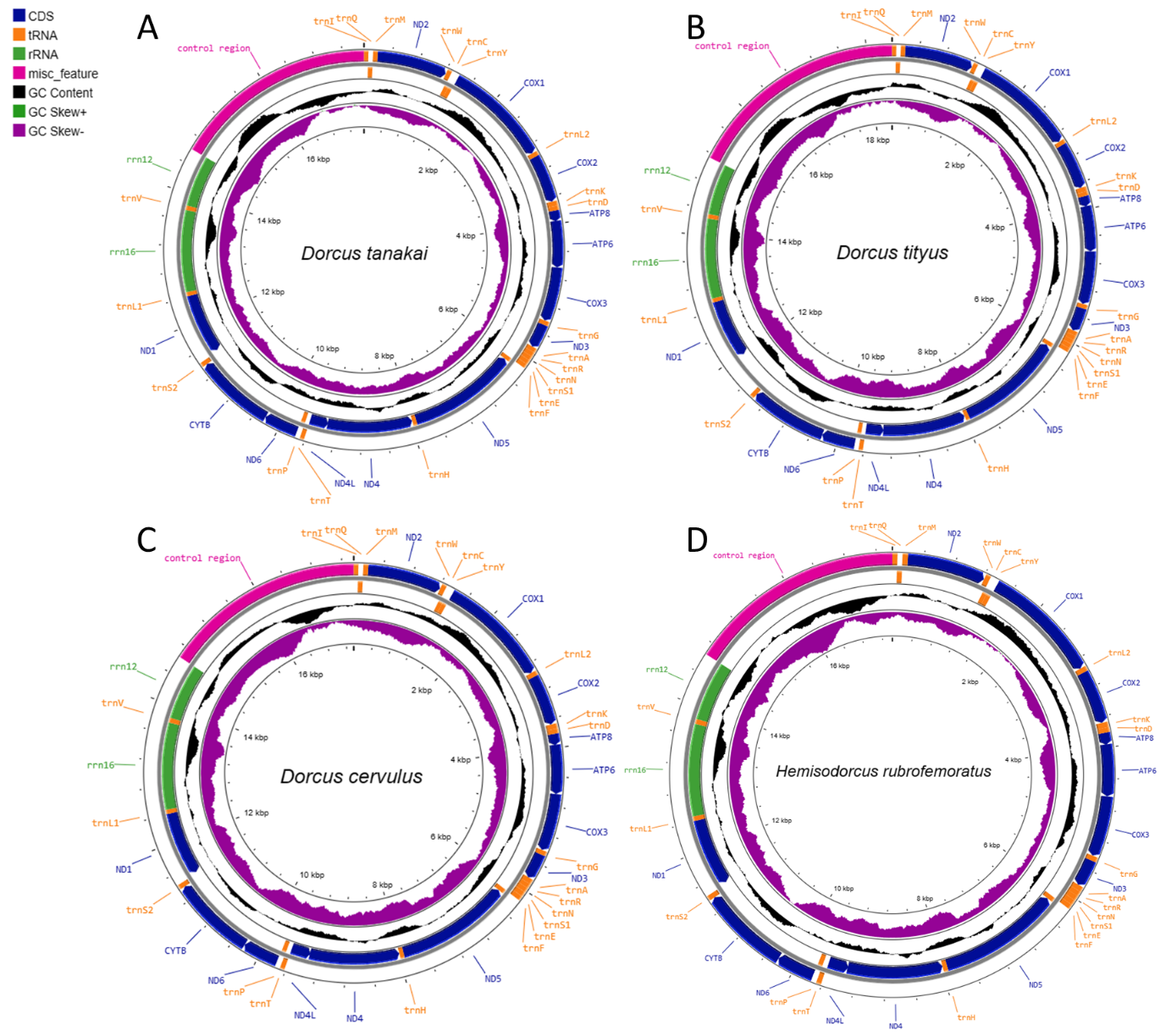


Fig. S3. Completely sequenced mitochondrial genome of *Dorcus* specimens including all the genes and control region. A: *Dorcus* *tanakai* (Accession No. OL944347), B: *Dorcus* *tityus* (Accession No. OL944348), C: *Dorcus cervulus* (Accession No. OL944356), D: *Dorcus rubrofemoratus* (Accession No. OL944354).


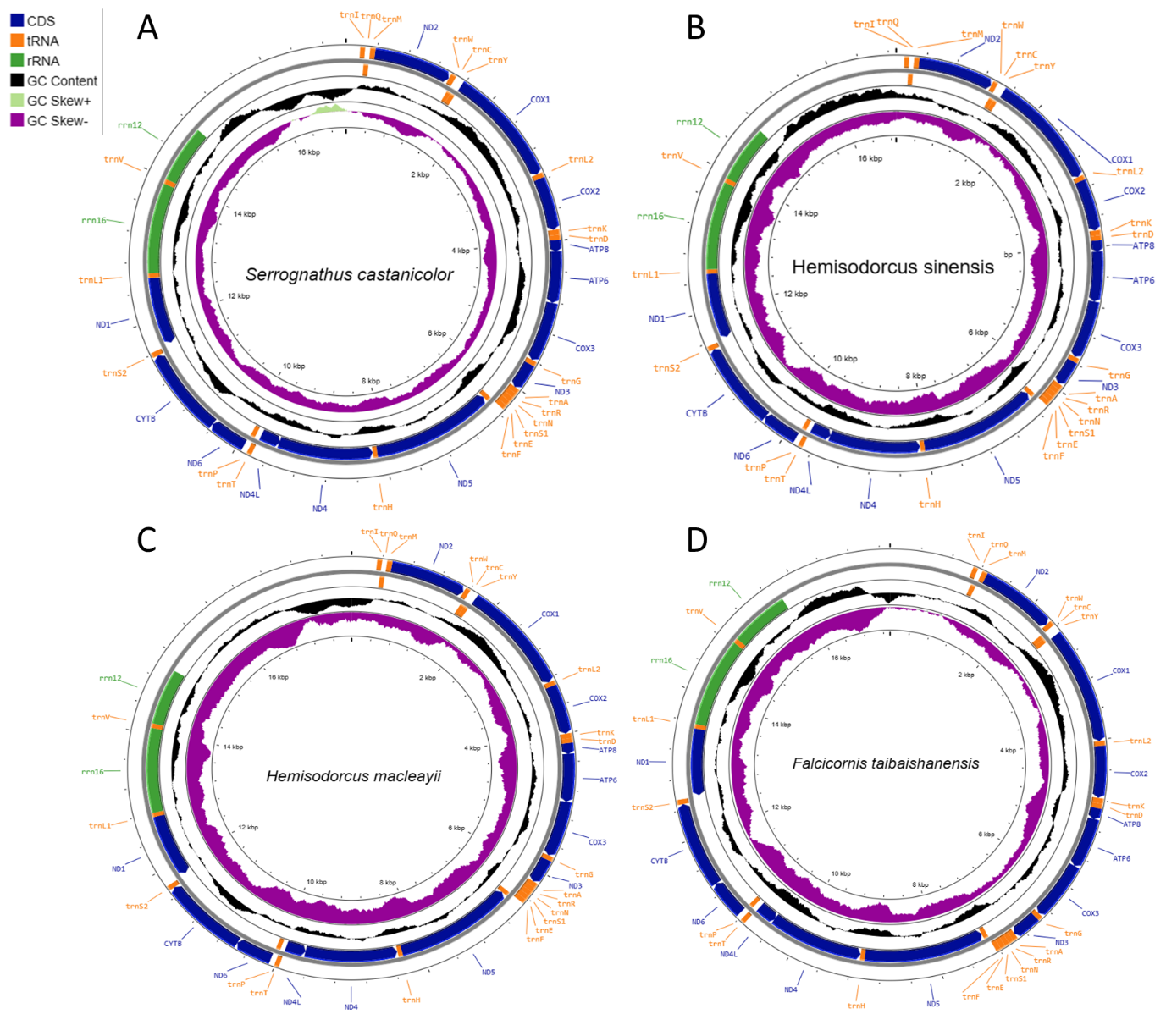


Fig. S4. Partially complete sequenced mitochondrial genome of *Dorcus* specimens having all the genes but missing control region. A: *Serrognathus castanicolor* (Accession No. OL944357), B: *Hemisodorcus sinensis* (Accession No. OL944355), C: *Hemisodorcus macleayii* (Accession No. OL944353), D: *Falcicornis taibaishanensis* (Accession No. OL944349).


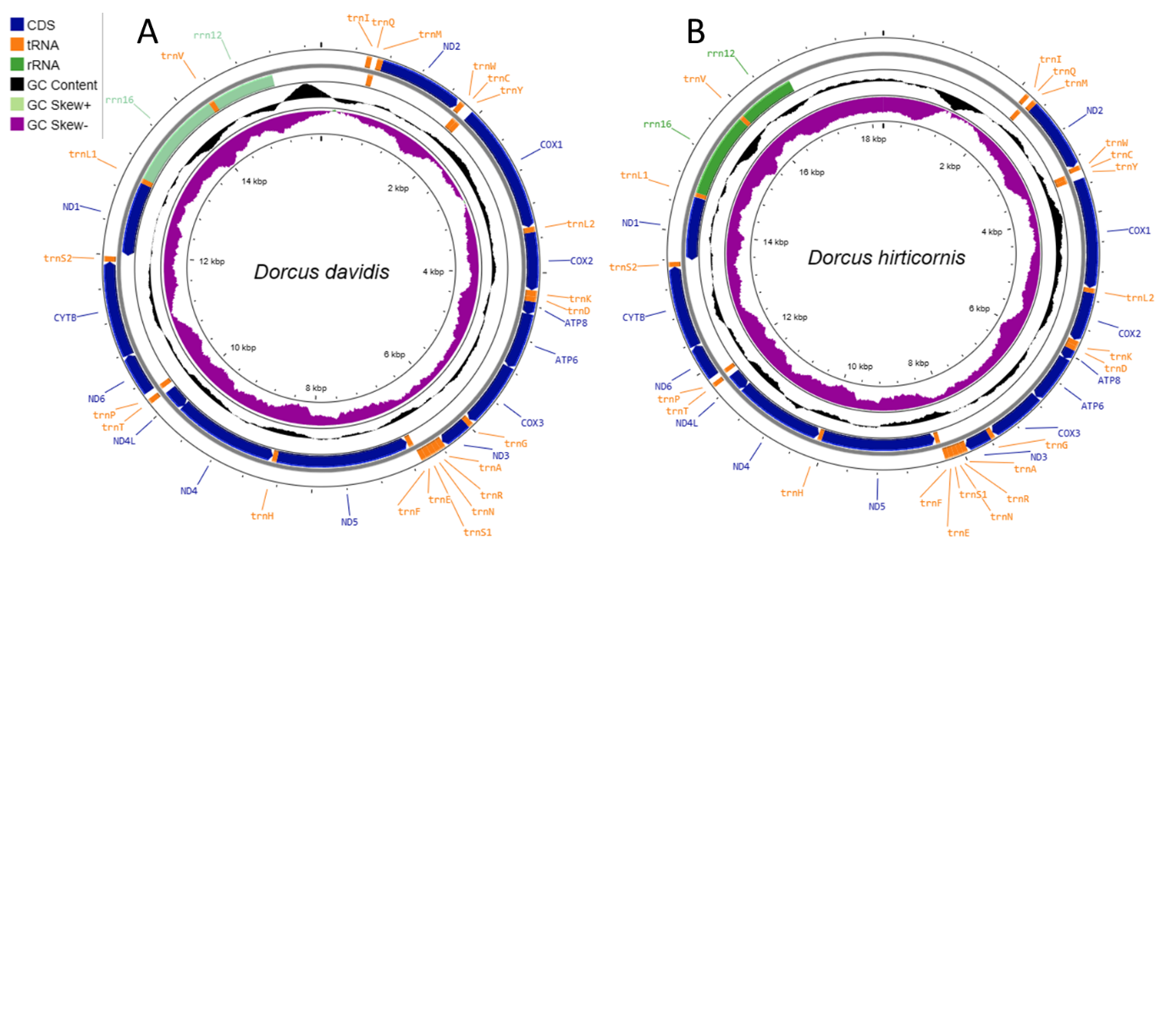


Fig. S5. Partially complete sequenced mitochondrial genome of *Dorcus* specimens having all the genes but missing control region. A: *Dorcus davidis* (Accession No. OL944343), B: *Dorcus hirticornis* (Accession No. OL944358).


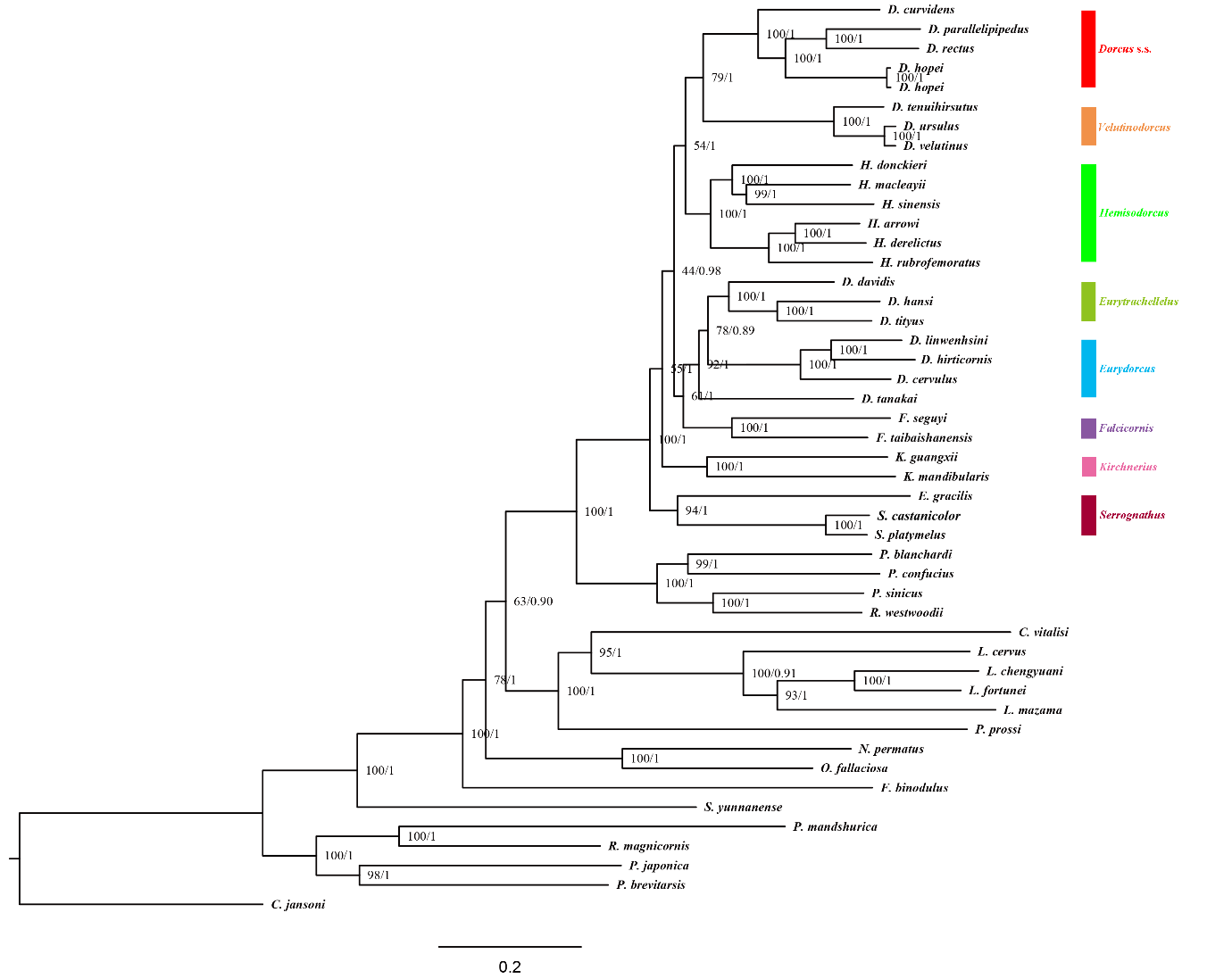


Fig.S6 Maximum likelihood (ML) method and Bayesian inferences (BI) based phylogenetic reconstruction of Eastern Asian *Dorcus* stag beetles using 13 protein-coding genes (PCGs).


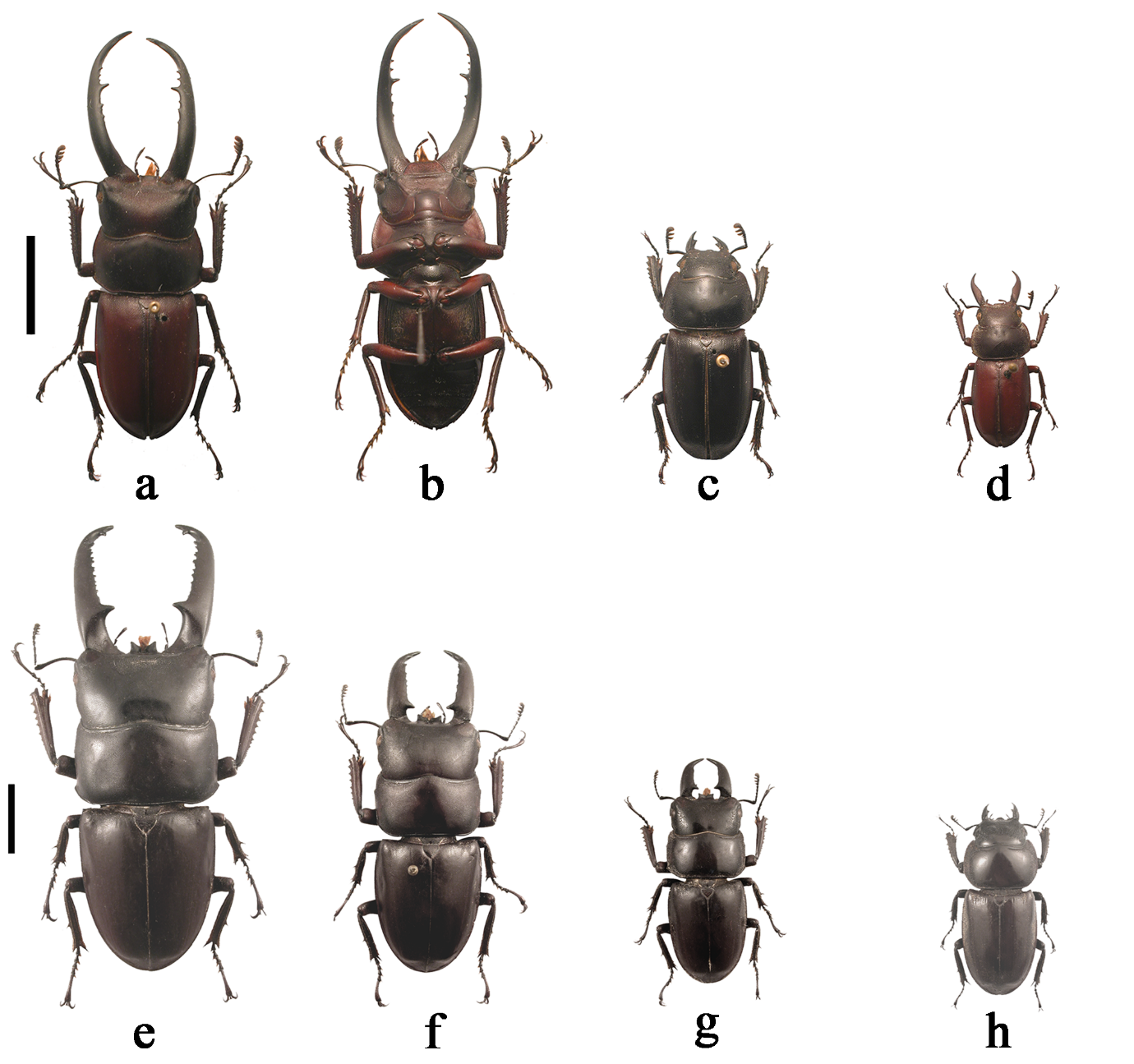


Fig. S7 a-d: Habitus of *Epidorcus gracilis*. e-h: Habitus of *Serrognathus castanicolor*. Scale, 10mm.


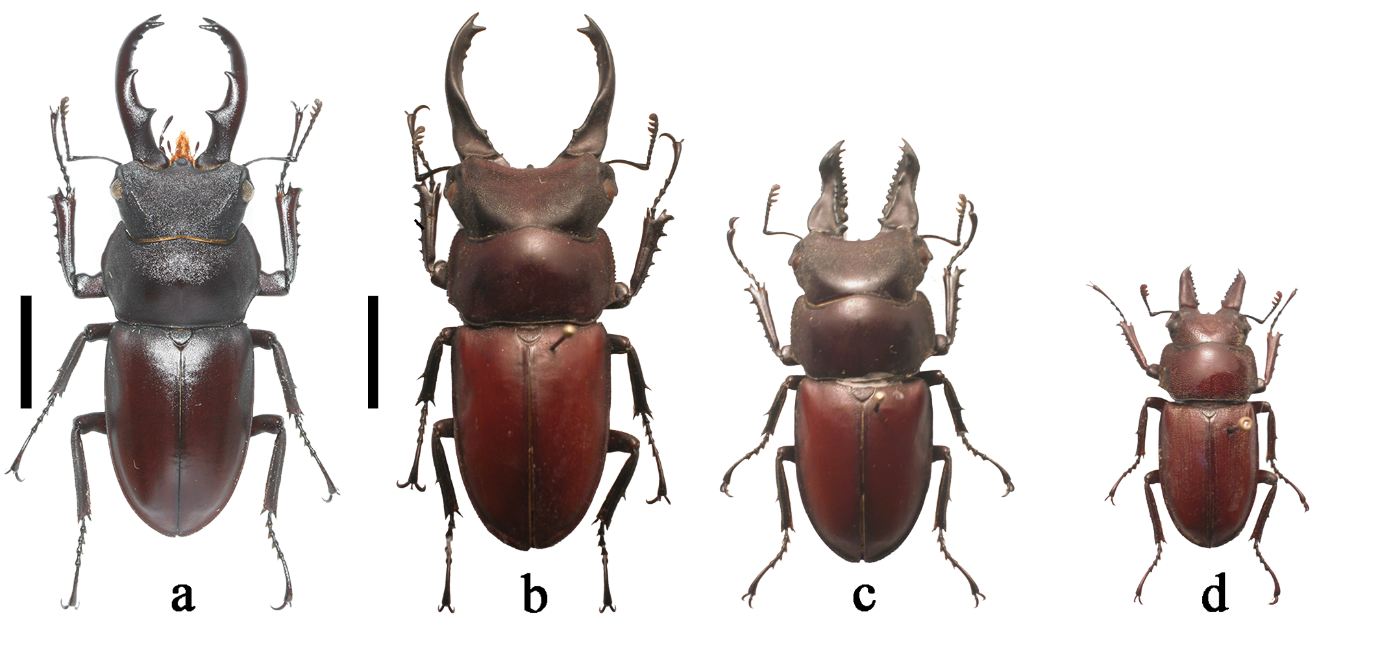


Fig. S8. a: Habitus of *Kirchnerius guangxii*. b-d: Habitus of *Kirchnerius mandibularis*. Scale, 10mm.


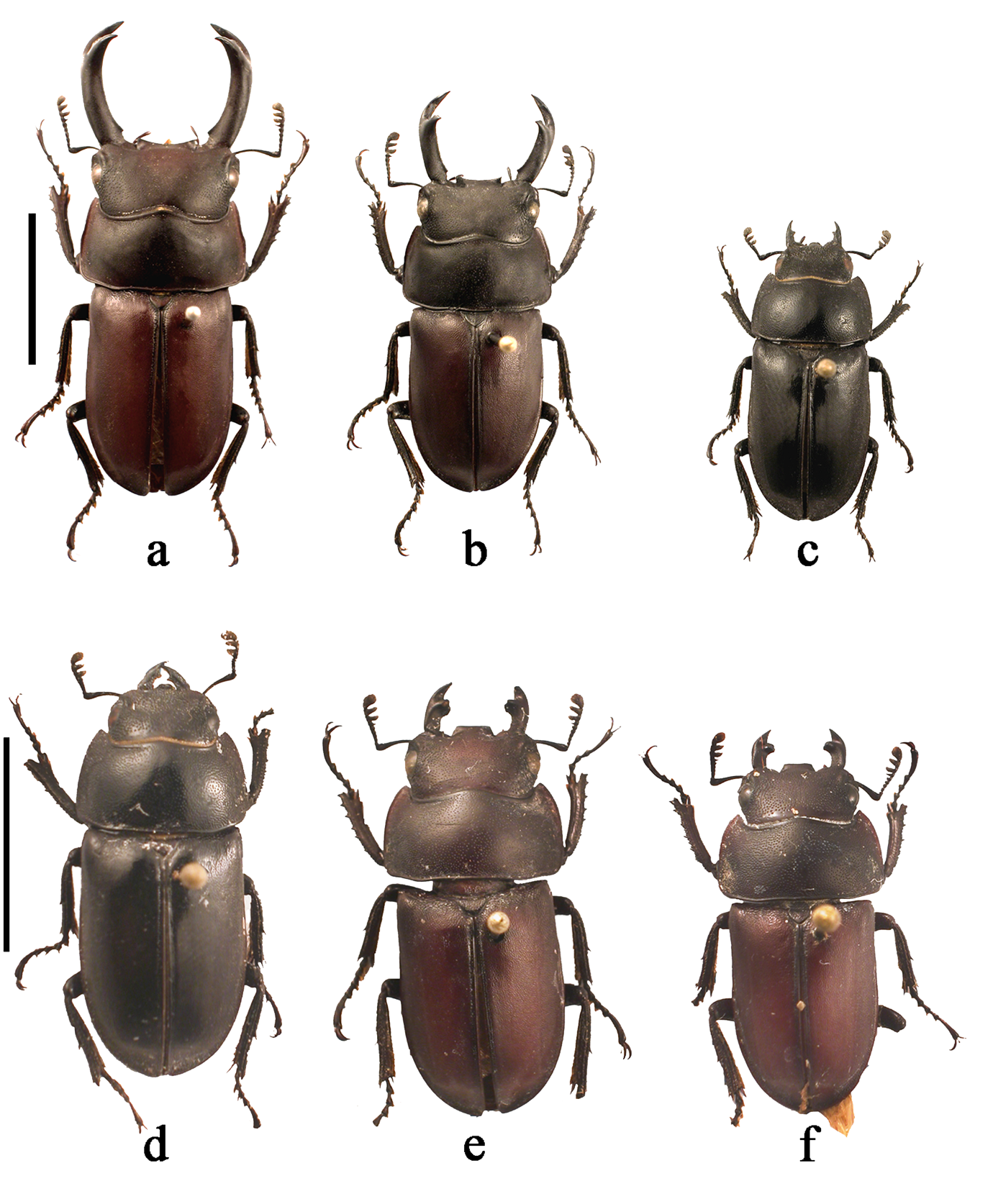


Fig. S9 a-c: Habitus of *Falcicornis seguyi.* d-f: Habitus of *Falcicornis taibaishanensis*. Scale, 10mm.


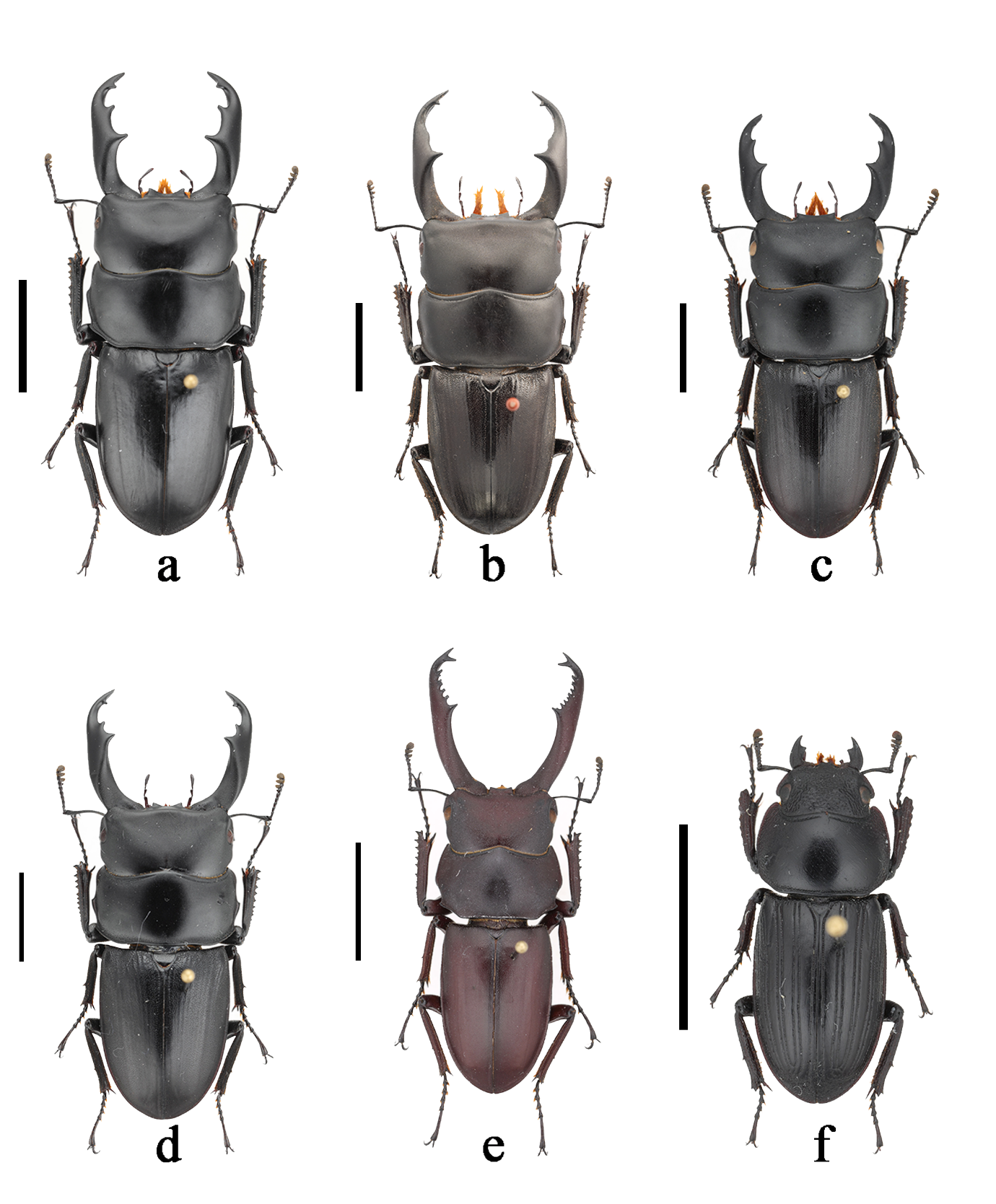


Fig. S10 a: Habitus of *Dorcus linwenhsini*. b: Habitus of *Serrognathus hirticornis*. c-d: Habitus of *Serrognathus cervulus*. e-f: Habitus of *Dorcus tanakai*. Scale, 10mm.


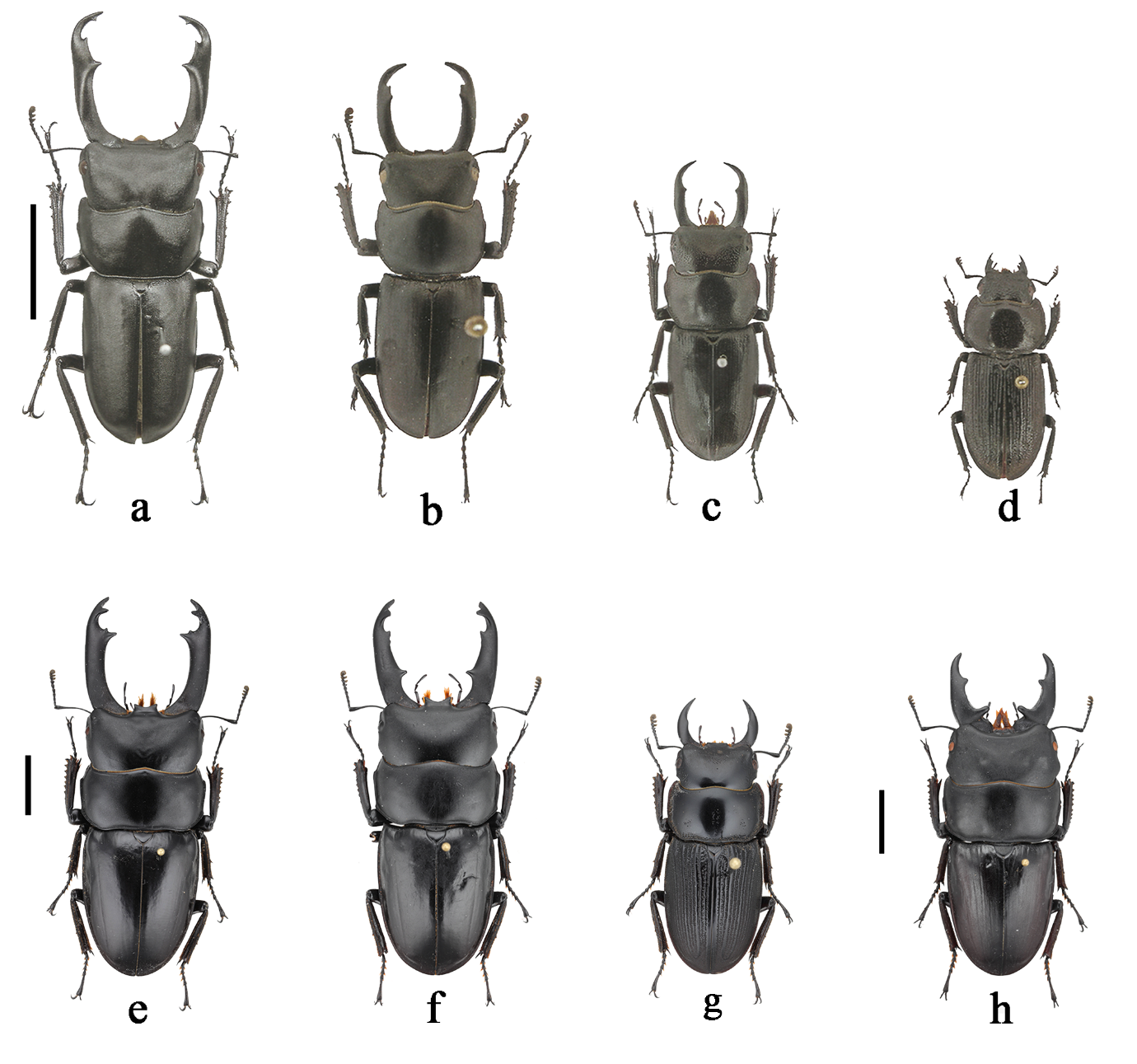


Fig. S11 a-d: Habitus of *Dorcus davidi.* e-g: Habitus of *Dorcus tityus*. h: Habitus of *Dorcus hansi*.. Scale, 10mm.


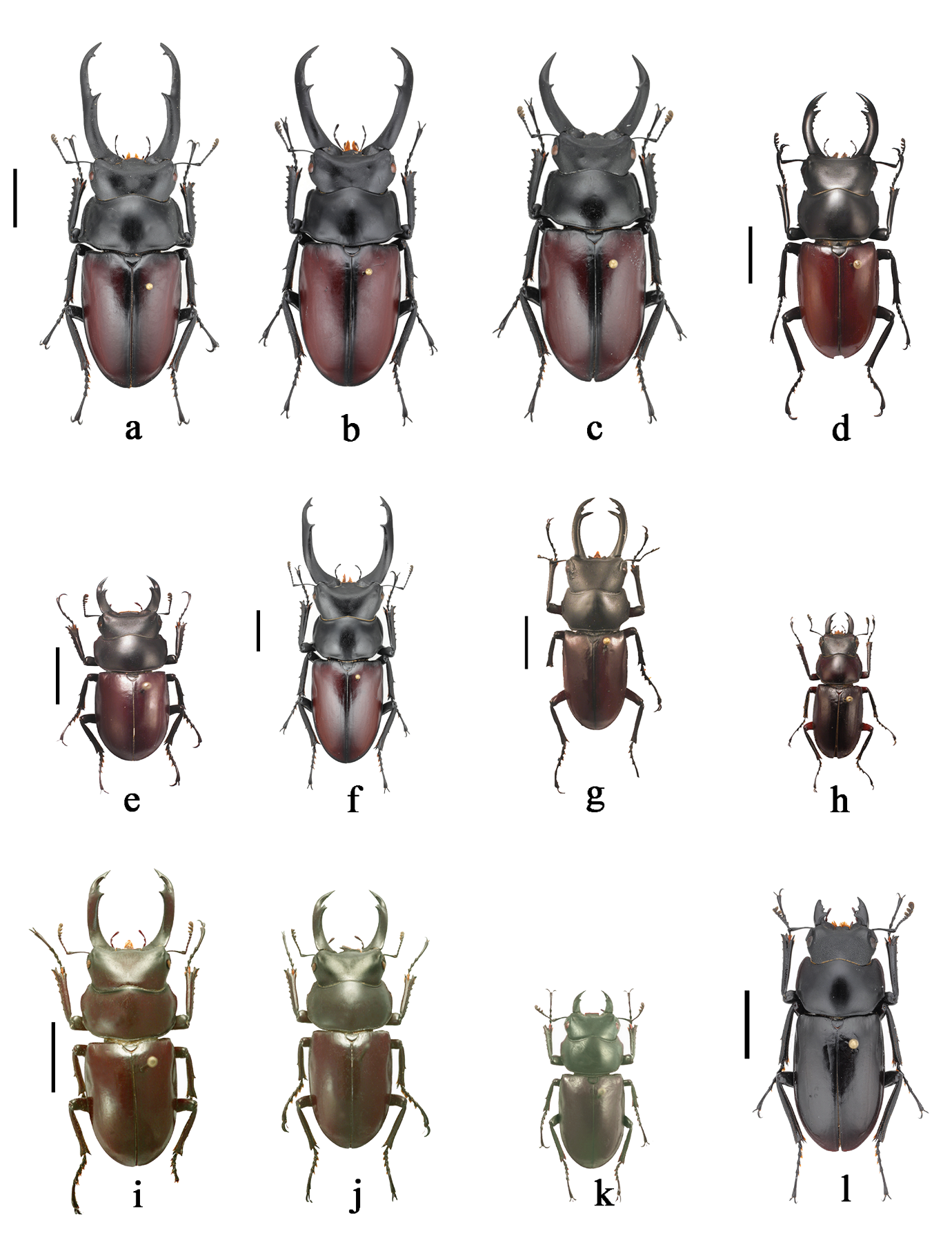


Fig. S12 **.** a-c: Habitus of *Hemisodorcus donckieri*. d-e: Habitus of *Hemisodorcus arrowi*. f: Habitus of *Hemisodorcus macleayii*. g-h: Habitus of *Hemisodorcus rubrofemoratus.* i-k: Habitus of *Hemisodorcus sinensis*. l: Habitus of *Hemisodorcus derelictus.* Scale, 10mm.


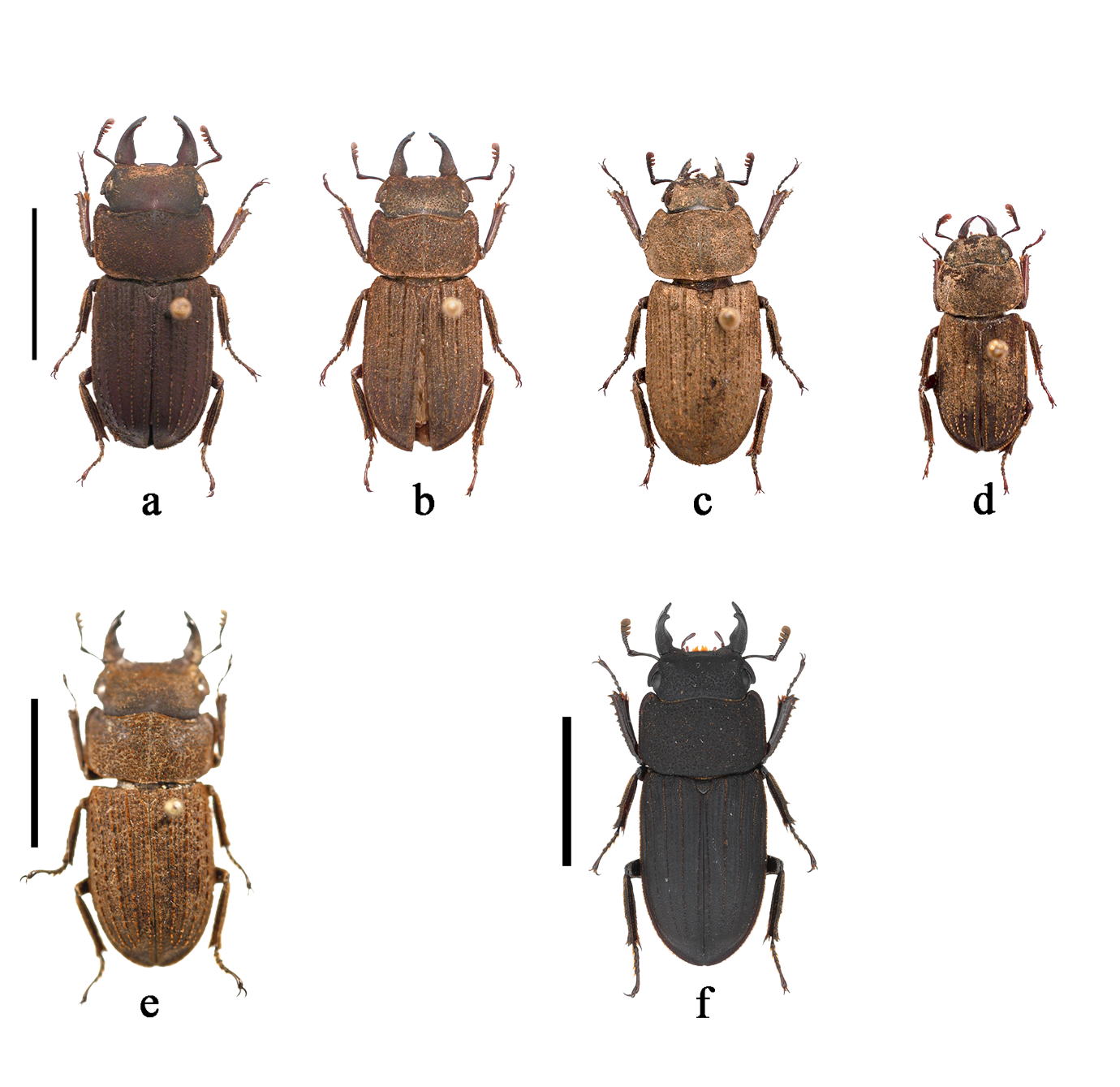


Fig. S13 a-d: Habitus of *Dorcus velutinus*. e: Habitus of *Dorcus ursulus*. f: Habitus of *Dorcus tenuihirsutus.* Scale, 10mm.


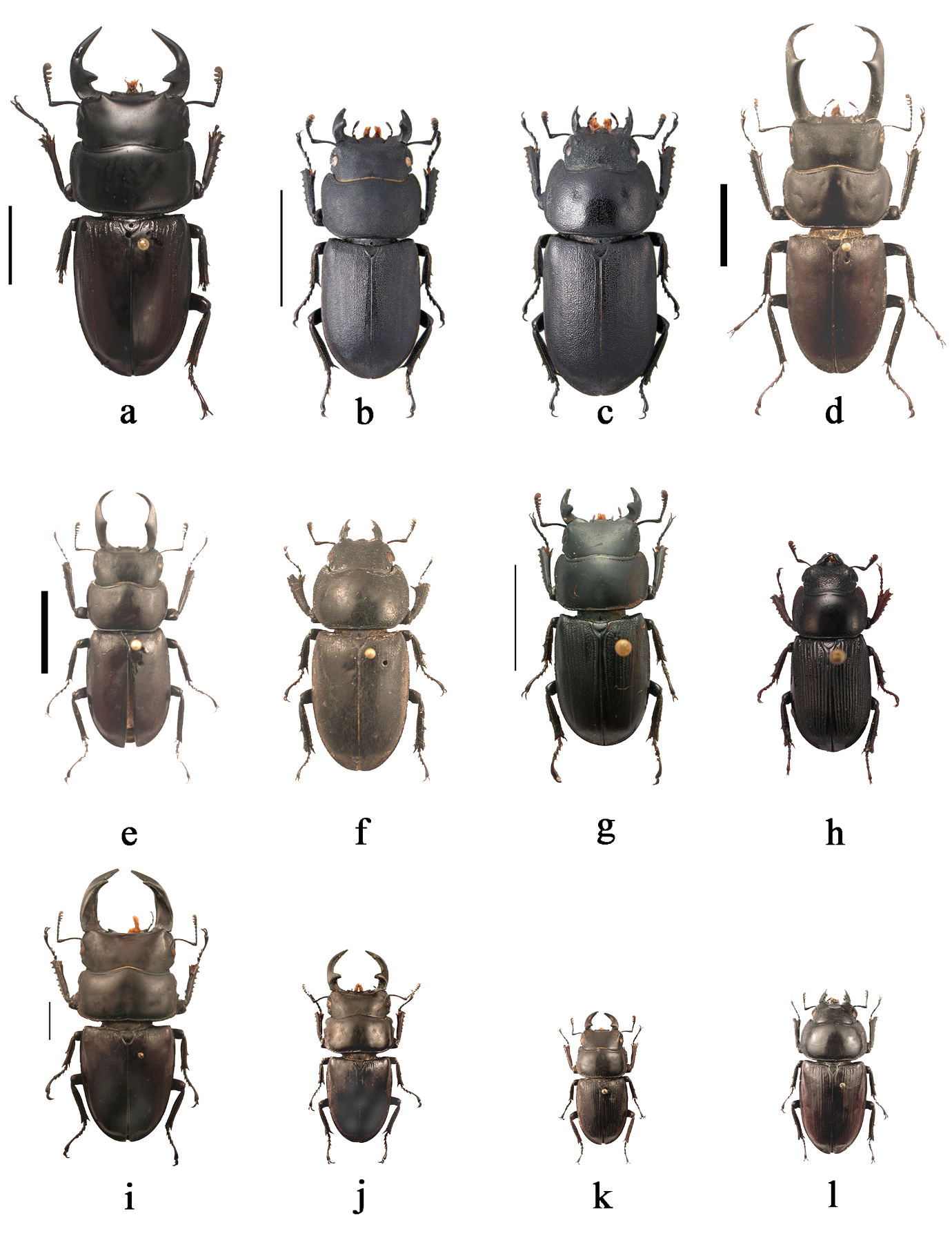


Fig. S14 a: Habitus of *Dorcus curvidens*. b-c: Habitus of *Dorcus parallelipedus*. d-f: Habitus of *Dorcus rectus.* g-h: Habitus of *Dorcus hopei.* i-l: Habitus of *Dorcus hopei.* Scale, 10mm. (see figure b-c in after Bartolozzi et al (2014 , fig. 16-17)).

References

1. Simon C, Frati F, Beckenbach A, Crespi B, Liu H, Flook P. Evolution, weighting, and phylogenetic utility of mitochondrial gene sequences and a compilation of conserved polymerase chain reaction primers. Annals of the entomological Society of America. 1994;87(6):651-701.

2. Balke M, Watts CH, Cooper SJ, Humphreys WF, Vogler AP. A highly modified stygobiont diving beetle of the genus Copelatus (Coleoptera, Dytiscidae): taxonomy and cladistic analysis based on mitochondrial DNA sequences. Systematic entomology. 2004;29(1):59-67.

3. Hosoya T, Araya K. Phylogeny of Japanese stag beetles (Coleoptera: Lucanidae) inferred from 16S mtrRNA gene sequences, with reference to the evolution of sexual dimorphism of mandibles. Zoological Science. 2005;22(12):1305-18.
